# Synthesis and Evaluation of Poly(3-hydroxypropyl Ethylene-imine) and Its Blends with Chitosan Forming Novel Elastic Films for Delivery of Haloperidol

**DOI:** 10.3390/pharmaceutics14122671

**Published:** 2022-11-30

**Authors:** Sitthiphong Soradech, Pattarawadee Kengkwasingh, Adrian C. Williams, Vitaliy V. Khutoryanskiy

**Affiliations:** 1 Reading School of Pharmacy, University of Reading, Whiteknights, Reading RG6 6DX, UK; 2 Expert Centre of Innovative Herbal Products, Thailand Institute of Scientific and Technological Research, Pathum Thani 12120, Thailand

**Keywords:** chitosan, poly(3-hydroxypropyl ethyleneimine), polymer blend, elastic films, miscibility, haloperidol

## Abstract

This study aimed to develop novel elastic films based on chitosan and poly(3-hydroxypropyl ethyleneimine) or P3HPEI for the rapid delivery of haloperidol. P3HPEI was synthesized using a nucleophilic substitution reaction of linear polyethyleneimine (L-PEI) with 3-bromo-1-propanol. ^1^H-NMR and FTIR spectroscopies confirmed the successful conversion of L-PEI to P3HPEI, and the physicochemical properties and cytotoxicity of P3HPEI were investigated. P3HPEI had good solubility in water and was significantly less toxic than the parent L-PEI. It had a low glass transition temperature (T_g_ = −38.6 °C). Consequently, this new polymer was blended with chitosan to improve mechanical properties, and these materials were used for the rapid delivery of haloperidol. Films were prepared by casting from aqueous solutions and then evaporating the solvent. The miscibility of polymers, mechanical properties of blend films, and drug release profiles from these formulations were investigated. The blends of chitosan and P3HPEI were miscible in the solid state and the inclusion of P3HPEI improved the mechanical properties of the films, producing more elastic materials. A 35:65 (%*w/w*) blend of chitosan–P3HPEI provided the optimum glass transition temperature for transmucosal drug delivery and so was selected for further investigation with haloperidol, which was chosen as a model hydrophobic drug. Microscopic and X-ray diffractogram (XRD) data indicated that the solubility of the drug in the films was ~1.5%. The inclusion of the hydrophilic polymer P3HPEI allowed rapid drug release within ~30 min, after which films disintegrated, demonstrating that the formulations are suitable for application to mucosal surfaces, such as in buccal drug delivery. Higher release with increasing drug loading allows flexible dosing. Blending P3HPEI with chitosan thus allows the selection of desirable physicochemical and mechanical properties of the films for delivery of haloperidol as a poorly water-soluble drug.

## 1. Introduction

Chitosan, a natural cationic polysaccharide [1], is widely used in numerous applications, including drug delivery systems, artificial skin, cosmetics, nutrition, and food additives, due to its biocompatibility, biodegradability, adhesivity, antimicrobial properties, and film-forming abilities [2,3,4]. Further, chitosan has been reported to improve skin penetration and wound healing by increasing the function of inflammatory and repair cells [5,6,7] and has been used to control drug release for transdermal [6] or transmucosal [8] delivery. However, chitosan-based films have some limitations, including low elasticity and brittleness [9]. In addition, the glass transition temperature (T_g_) of film-forming polymers affects the drug release profile, since temperatures above T_g_ enable polymer chain movement, which facilitates drug release [10]. The physiological temperature of mucosal membranes is ~35–37 °C and external skin temperature is ~32 °C [11], whereas chitosan has a high glass transition temperature (~131 °C) [9], which can restrict drug release from films applied to these biological surfaces.

The poor mechanical properties and high glass transition temperature of chitosan can be moderated by blending with other water-soluble polymers [12]. Blending provides a simple and low-cost approach to design materials with tailored properties. For example, improved mechanical and mucoadhesive properties of chitosan-based films were achieved by combining cellulose ethers and chitosan [13]. Chitosan has also been blended with poly(N-vinyl pyrrolidone) [14], poly(ethylene oxide) [15], and poly(vinyl alcohol) [16] to improve physicochemical properties. Luo et al. [12] developed elastic films using chitosan–hydroxyethylcellulose blends (HEC), whereas Abilova et al. [9] developed films using chitosan and poly(2-ethyl-2-oxazoline) (PEOZ) blends. The T_g_ of films based on chitosan and PEOZ decreased from 131 to 63 °C, although the mechanical properties of the resultant films were also compromised as the proportion of PEOZ increased [9].

Linear polyethyleneimine (L-PEI) is a cationic polymer composed of two aliphatic carbon spacer groups (-CH_2_CH_2_-) and secondary amine groups in each repeating unit [17]. It can be synthesized by hydrolysis of poly(2-ethyl-2-oxazoline) to remove all amide side chains [18,19]. Generally, L-PEI is semi-crystalline [20,21] with a glass transition temperature ~−29.5 °C [22] and, therefore, appears to be a suitable candidate to blend with chitosan in order to improve film mechanical properties and drug release profiles. However, L-PEI only dissolves in water at high temperatures [20], forms a gel at room temperature [23], and has been shown to cause cytotoxicity [22,24]. The solubility and toxicity of L-PEI is naturally a significant concern when considering its use in pharmaceutical and biomedical applications [25]. Chemical modifications of L-PEI is one approach to increasing solubility in water and decreasing its toxicity [26]. Patil et al. [26] used nucleophilic substitution to synthesize hydroxyethyl-substituted linear polyethylenimine (HELPEI) for the delivery of siRNA therapeutics; the cytotoxicity of HELPEI on human bronchial epithelial cells decreased as the degree of substitution increased. Here, we selected poly(3-hydroxypropyl ethyleneimine) (P3HPEI) as a suitable candidate to blend with chitosan to produce films to deliver haloperidol and report, for the first time, its synthesis using nucleophilic substitution reaction of linear polyethyleneimine with 3-bromo-1-propanol.

Haloperidol (HP), an antipsychotic drug, is associated with the side effect of drug-induced extrapyramidal syndrome (EPS) in conventional monotherapy [27]. It is poorly water soluble and is commonly formulated as a solution for oral administration or injections and as tablets [18]. The average oral dose of haloperidol ranges from 0.5 to 30 mg per day [28]. Further, HP is a BCS class 2 drug, characterized by low solubility but high permeability [29] and has poor oral bioavailability (59%) [28]. Consequently, Samanta et al. [27] developed HP-loaded matrix dispersion films with Eudragit NE 30D as a controlled release transdermal dosage form. Gidla et al. [29] developed HP-loaded buccal films to provide rapid onset of drug action, with improved patient compatibility and without requiring swallowing. However, HP-loaded films using Eudragit NE 30D [27] or hydroxypropyl methylcellulose (HPMC) [29] have some limitations due to the high glass transition temperature of these polymers, which can restrict drug release from the films when applied to skin or mucosal surface. Therefore, loading HP in polymer blends where the glass transition temperature (T_g_) has been optimized to below the temperature of mucosal membranes (~35–37 °C) or external skin (32 °C) [11] can improve drug release into mucosal tissue and also improve the mechanical properties of films.

Here, we aimed to develop novel elastic films based on chitosan and poly(3-hydroxypropyl ethyleneimine) or P3HPEI for the rapid buccal delivery of haloperidol. P3HPEI was synthesized as a novel water-soluble polymer for pharmaceutical and biomedical applications. The physicochemical properties and cytotoxicity of this new material were assessed before it was blended with chitosan to fabricate elastic films for the rapid delivery of haloperidol, chosen as a model poorly water-soluble drug. Miscibility between polymers and the mechanical properties of blend films are reported. An optimal composition of chitosan and P3HPEI blend was then selected for drug incorporation and release studies.

## 2. Materials and Methods

### 2.1. Materials

High molecular weight chitosan (CHI, MW∼310–375 kDa, degree of deacetylation: 75–85%), poly(2-ethyl-2-oxazoline) (PEOZ, MW∼50 kDa, PDI 3–4), 3-bromo-1-propanol, hydrochloric acid solution, fluorescein isothiocyanate (FITC), and haloperidol were purchased from Merck (Gillingham, UK), while phosphate-buffered saline (PBS) tablets and sodium hydroxide were from Fisher Chemicals (Fisher Scientific, Leicestershire, UK). All other chemicals were of analytical grade and used without further purification.

### 2.2. Synthesis of Linear Poly(ethyleneimine) (L-PEI)

L-PEI was synthesized by hydrolysis of poly(2-ethyl-2-oxazoline), as described in our previous study [17]. Briefly, 10 g of poly(2-ethyl-2-oxazolines) (PEOZ) was dissolved in 100 mL of 18.0% (*w/w*) hydrochloric acid and then refluxed at 100 °C for 14 h to remove all amide groups in the side chains. The L-PEI solution was then diluted with cold deionized water (500 mL). Cold aqueous sodium hydroxide (4M) was added dropwise to the suspension until the polymer dissolved, with further addition of 4 M sodium hydroxide, the base form of L-PEI precipitated at pH 10–11 [30]. The precipitate was recovered by using a vacuum filtration, washed with deionized water, and re-precipitated twice before drying under vacuum oven at 25–30 °C for several days to obtain L-PEI as a white powder yielding 3.8 g (89%).

### 2.3. Synthesis of Poly(3-hydroxypropyl ethyleneimine) (P3HPEI)

L-PEI (0.02 moles per repeating unit, 1.0 g) was dissolved in absolute ethanol (60 mL) in a three-necked round-bottom flask before 0.06 moles of 3-bromo-1-propanol (5.3 mL) were added. As a proton abstractor, 0.06 moles of potassium carbonate (8.1 g) were then added before the reaction mixture was refluxed at 78 °C for 24 h; the reaction scheme is shown in Figure S1. After centrifugation of the reaction mixture, the supernatant was collected and evaporated using a rotary evaporator at 40 °C and 280 rpm. The resulting mixture was diluted with deionized water and purified using dialysis with a cellulose-based membrane (MWCO = 3.5 kDa) at room temperature. P3HPEI was recovered as dry residue (77% yield) by freeze-drying (−56.5 °C and 0.25 hPa) for several days.

### 2.4. Preparation of Films

**Figure 1:**
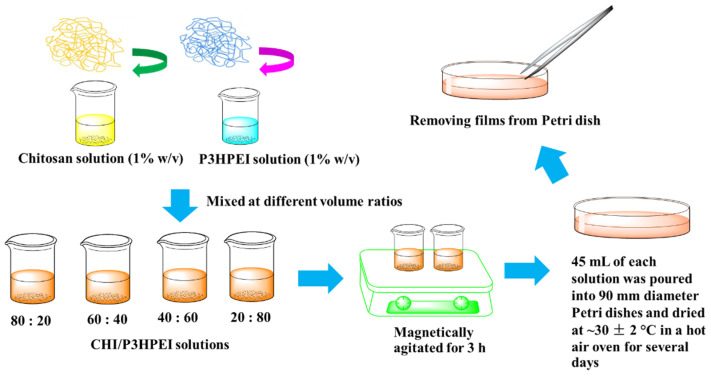
Scheme of CHI/P3HPEI films preparation.

Chitosan (CHI) films and their blends with P3HPEI were prepared by casting and solvent evaporation, as schematically shown in Figure 1. Initially, 1.0% *w/v* aqueous solutions of CHI and P3HPEI were prepared at room temperature; CHI solution (pH~2.0) was prepared in 0.1 M hydrochloric acid by stirring magnetically for 24 h, whereas P3HPEI solutions (pH~6.8) were prepared in deionized water and allowed to stir continuously for 1 h. The polymer solutions were mixed at different volume ratios, denoted as CHI 100:0 and CHI/P3HPEI: 80:20, 60:40, 40:60 and 20:80. The pH of the combined solutions was in the range of 3.0–4.0. All CHI/P3HPEI solutions were magnetically agitated for 3 h to ensure a homogeneous mixture formed. Then, 45 mL of each solution was poured into 90 mm diameter Petri dishes and dried at ~30 ± 2 °C in a hot air oven for several days until dry films formed.

### 2.5. Preparation of Haloperidol-Loaded Films

A stock solution of haloperidol (5 mg/mL) was prepared by initially dissolving 50 mg of the drug in 5 mL of absolute ethanol. Subsequently, the total volume of haloperidol solution was adjusted to 10 mL. Aliquots were added to 10 mL of 1.0% CHI/P3HPEI solution to obtain 1.0–5.0% drug loading, followed by stirring for 2 h before casting and drying into films, as described above, but instead using 10 mL of solution decanted into 35 mm diameter Petri dishes. The content of haloperidol in each film of 35 mm in diameter was 1.25 to 5.00 mg.

### 2.6. Characterization of Polymers and Films

#### 2.6.1. ^1^H-Nuclear Magnetic Resonance Spectroscopy (^1^H-NMR)

Approximately 20 mg of dried PEOZ or L-PEI was dissolved in 1 mL methanol-d_4_, whereas P3HPEI was dissolved in 1 mL D_2_O, before the samples were added into NMR tubes and analyzed using a 400 MHz ULTRASHIELD PLUSTM B-ACS 60 spectrometer (Bruker, UK). Data processing used MestReNova software. The degree of substitution (DS) of P3HPEI was determined using peak integration, according to Equation (1):

(1) $$\%\;\mathrm{DS}=\;\frac{ʃ\mathrm{Peak}\;b/n_{b\;}}{ʃ\mathrm{Peak}\;a/n_{a}}\times 100$$

where peak a corresponds to CH_2_CH_2_ on the main backbone adjacent to nitrogen, Peak b corresponds to CH_2_ on side group, n_a_ is the number of protons in CH_2_CH_2_ on the main backbone adjacent to nitrogen, and n_b_ is number of protons in CH_2_ on the side group.

#### 2.6.2. Fourier Transformed Infrared (FTIR) Spectroscopy

Polymer and film samples were scanned from 4000 to 400 cm^−1^ at a resolution of 4 cm^−1^. Data were processed from the average of six scans per spectrum generated by the Nicolet iS5-iD5 ATR FT-IR spectrometer (Thermo Scientific, Leicestershire, UK).

#### 2.6.3. Differential Scanning Calorimetry

Thermal analysis used a Q100 DSC (TA Instruments, Hüllhorst, Germany). Polymer and film samples (~3–5 mg) were loaded into pierced Tzero aluminum pans. The thermal behavior of each sample was investigated in a nitrogen atmosphere with a heating/cooling rate of 10 °C/min (−70 to 180 °C). The glass transition temperatures (T_g_) of polymers were determined from the second heating cycle.

#### 2.6.4. Thermogravimetric Analysis (TGA)

Thermogravimetric analysis used a Q50 TGA analyzer (TA Instruments, Crawley, UK) over the range between 20 and 600 °C heating at 10 °C/min under a nitrogen atmosphere. Prior to analysis, the films dried in a vacuum oven (as above) and were placed in a desiccator over dry silica gel for 3 days. Moisture content in each film was determined from the weight loss corresponding to the first step weight loss in their TGA curves (up to ~150 °C).

#### 2.6.5. Powder X-ray Diffractrometry (PXRD)

Dry polymers or films were placed on a silica slide and analyzed with a Bruker D8 ADVANCE PXRD equipped with a LynxEye detector and monochromatic Cu Kα_1_ radiation (λ = 1.5406 Å). Samples were rotated at 30 rpm and data collected over an angular range (2θ) of 5–60° for 1 h, with a step of 0.05° (2θ), and count time of 1.2 s. The data were analyzed using Origin software.

#### 2.6.6. Film Thickness

Polymer film thicknesses were measured with a digital micrometer (Mitutoyo, Kawasaki, Japan) with 0.001 mm resolution. Measurements were taken at several points of the film before the mean values ± SD were calculated (Table S1).

#### 2.6.7. Scanning Electron Microscopy (SEM)

SEM experiments used an FEI Quanta 600 FEG Environmental Scanning Electron Microscope instrument (FEI UK Ltd., Cambridge, UK), with an acceleration voltage of 20 kV. Images were taken from the fracture surface of the materials, which were first frozen in liquid nitrogen and coated with gold sputter to facilitate high resolution imaging.

#### 2.6.8. Fluorescence Microscopy

The fluorescein isothiocyanate (FITC)-labelled chitosan was synthesized, according to Cook et al. [31]. Briefly, dehydrated methanol (100 mL) and FITC (2 mg/mL in methanol, 50 mL) were added to a chitosan solution (1% *w/v* in 0.1 M hydrochloric acid, 100 mL). The reaction was carried out in the dark at room temperature for 3 h before precipitation in NaOH (0.1 M, 1 L). The resulting precipitate was filtered and dialyzed in deionized water (4 L, replaced daily) until FITC was not detected in the dialysate, before the product was freeze-dried. Then, films were prepared as described before (see Section 2.4), albeit using 10 mL of each solution poured into 30 mm diameter Petri dishes before drying. The morphology of the films was analyzed using fluorescence microscopy at 20× magnification.

#### 2.6.9. Polarized Light Microscopy

Films were examined for the presence of haloperidol crystals using a polarized light microscope (Mettler Toledo FP90, Germany) at 20× magnification; images were analyzed using Infinity 1 camera (Lumenera Corporation, Nepean, ON, Canada).

#### 2.6.10. Mechanical Properties

The mechanical properties of the films—puncture strength, elongation at puncture, and modulus at puncture—were determined using a TA.XT Plus Texture Analyser (Stable Micro Systems Ltd., Godalming, UK) in compression mode, adapted from our previous studies [9,32,33]. Square of film samples (30 mm × 30 mm) was fixed between two plates with a cylindrical hole of 10 mm diameter (area of the sample holder hole: Ar_s_, 78.57 mm^2^) and compressed by the upper load 5 mm stainless steel spherical ball probe (P/5S) at 1.0 mm/s. The plate was stabilized to avoid movements using two pins. The measurements started once the probe was in contact with the sample surface and continued until each film sample broke. Test settings were: pre-test speed 2.0 mm/s; test speed 1.0 mm/s; post-test speed 10.0 mm/s; target mode distance; 5 mm; trigger force 0.049 N. The force required to puncture the films (N) was used to calculate the puncture strength by:

(2) $$\mathrm{Puncture}\;\mathrm{strength}\;=\;\frac{F_{\max}}{{\mathrm{Ar}}_{s}}$$

where F_max_ is the maximum applied force, Ar_s_ is area of the sample holder hole, with Ar_s_ = πr^2^, where r is the radius of the hole.

(3) $$\mathrm{Elongation}\;(\%)=\left(\frac{\sqrt{r^{2}+d^{2}}-r}{r}\right)\times 100$$

where r is the radius of the film exposed in the cylindrical hole of the film holder and d represents the displacement of the probe from the point of contact to point of puncture.

(4) $$\mathrm{Modulus}\;\mathrm{at}\;\mathrm{puncture}=\frac{\mathrm{Puncture}\;\mathrm{strength}}{\mathrm{Elongation}\;\left(\%\right)}$$

### 2.7. In Vitro Drug Release Study

Haloperidol release from films was assessed using a modified Franz diffusion cell (FDC), which was adapted from Samanta et al. [27]. The receptor compartment was filled with 20 mL of 20% PEG 400 in phosphate-buffered saline (pH = 7.4) to ensure sink conditions [27] and was stirred at 600 rpm at 37 °C throughout the experiment. Films containing 5.0, 2.5, and 1.25% haloperidol were placed between the donor and receptor compartments. 1 mL aliquots were taken from the receptor compartment at predetermined time intervals and replaced with 1 mL fresh receiver medium to maintain a constant volume. Drug release was monitored for 180 min, with the drug concentration determined spectrophotometrically at its respective wavelength. A standard calibration curve of haloperidol, ranging from 5–50 µg/mL, was prepared (Figure S10). The protocol used for the preparation of stock solution of haloperidol is described in Supplementary Materials. For each type of film, three replicates were performed.

### 2.8. Cytotoxicity Test

Polymer cytotoxicity was evaluated using an MTT assay. Briefly, L-PEI was dissolved in 95% ethanol and then diluted with Dulbecco’s modified eagle medium (DMEM) to obtain polymer concentrations between 5–5000 µg/mL, whereas PEOZ and P3HPEI were dissolved directly in Dulbecco’s modified eagle medium (DMEM) and then diluted with DMEM to prepare solutions with polymer concentrations ranging between 5–5000 µg/mL. Human dermal fibroblasts (ATCC CRL-2522) were seeded at 1 × 10^5^ cells/mL in a 96 well plate and allowed to attach overnight before being incubated with the polymer samples at 5, 50, 500, 1000, 2500, and 5000 µg/mL for 24 h. As a positive control, 10% DMSO (*v/v*) in DMEM was used, and for the negative control we used 10% fetal bovine serum in DMEM. Then, 100 µL of 3-(4,5-dimethylthiazol-2-yl)-2,5-diphenyl tetrazolium bromide (MTT) solution was added into each well before the plate was incubated at 37 °C in a CO_2_ incubator for 3 h. The amount of formazan produced was then quantified from the absorbance at 570 nm using a standard plate reader (Thermo Scientific™ Multiskan™ GO, Vantaa, Finland).

### 2.9. Statistical Analysis

Data are presented as mean values ± standard deviation (SD) for no fewer than three independent experiments. Student’s *t*-test and one-way ANOVA were used to determine the extent of any differences between samples.

## 3. Results

### 3.1. Synthesis and Evaluation of Poly(3-hydroxypropyl ethyleneimine)

**Figure 2:**
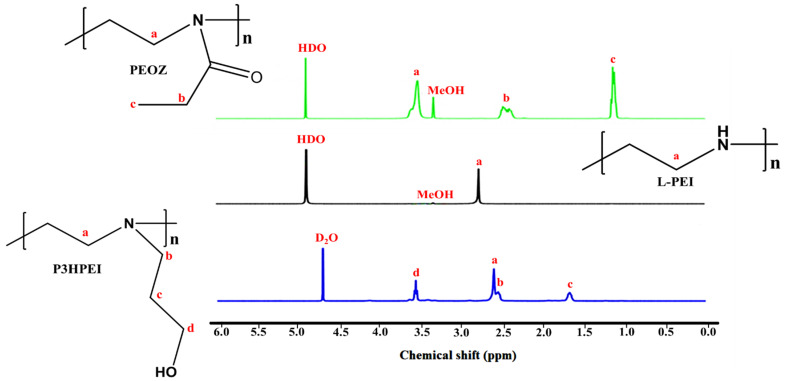
^1^H NMR spectra of PEOZ in MeOH-d_4_, L-PEI in MeOH-d_4_, and P3HPEI in D_2_O.

L-PEI was synthesized successfully by acidic hydrolysis of PEOZ to remove all amide groups from the side groups. The complete conversion to L-PEI was confirmed by ^1^H-NMR and FTIR spectroscopies. As illustrated in Figure 2, both PEOZ signals from the side groups, seen at 2.44 ppm and 1.13 ppm were eliminated, while the signal from the two methylene groups within the polymer backbone shifted to 2.75 ppm. FTIR (Figure S2) also confirmed the hydrolysis of the PEOZ amide groups through the loss of the amide carbonyl vibration at 1626 cm^−1^ and the appearance of new strong peaks ~1474 cm^−1^ and 3263 cm^−1^ due to the N-H vibration in PEI. The ^1^H-NMR and FTIR results from hydrolysis of PEOZ to obtain L-PEI correlated well with earlier reports [18,34].

The L-PEI was then alkylated via nucleophilic substitution reaction with 3-bromo-1-propanol in absolute ethanol, with potassium carbonate as a base. The obtained P3HPEI was also characterized using ^1^H-NMR and FTIR spectroscopies. The ^1^H NMR spectrum revealed four signals at approximately 2.65 ppm (signal a) due to -CH_2_CH_2_- in the polymer backbone, 2.52 ppm (signal b) assigned to the -CH_2_- in the side group adjacent to the backbone -CH_2_CH_2_-, 1.66 ppm (signal c) due to the -CH_2_- further down the side group, and 3.54 ppm (signal d) arising from the terminal -CH_2_- in the side group, adjacent to a hydroxyl group (-OH).

The degree of substitution (DS) of P3HPEI was determined by comparing the area under the peak from the methylene group on the side group to the area under the peak corresponding to the two methylene groups on the main backbone. The result indicated that the DS of P3HPEI was 97%, 99%, and 99%, as calculated based on signal b, c, and d, respectively. In addition, FTIR data (Figure S2) confirmed the successful synthesis of the hydroxypropyl substituted L-PEI (P3HPEI) by providing a broad absorption band at 3307 cm^−1^, assigned as an OH- stretching mode.

**Figure 3:**
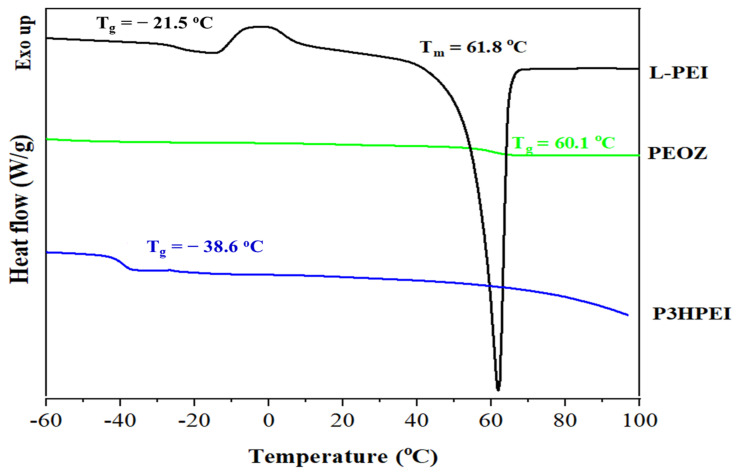
DSC thermograms of PEOZ, L-PEI, and P3HPEI.

Differential scanning calorimetry (DSC) and thermogravimetric analysis (TGA) characterized the thermal properties of PEOZ, L-PEI, and P3HPEI. DSC showed that PEOZ, L-PEI, and P3HPEI had glass transition temperatures (T_g_) of 60.1, −21.5, and −38.6 °C, respectively (Figure 3). Furthermore, the DSC thermogram of L-PEI gave a melting point of 61.8 °C, consistent with the literature [22,35]. The conversion of L-PEI to P3HPEI, with the inclusion of the side group containing a hydroxyl group, increases chain mobility and polymer flexibility, resulting in a change from a semi-crystalline to an essentially amorphous polymer; X-ray diffractometry confirmed that L-PEI is semi-crystalline, whereas the diffraction pattern from P3HPEI showed no evidence for crystallinity (Figure S3).

TGA was used to compare the thermal stability of P3HPEI to PEOZ and L-PEI (Figure S4). It is noticed that there are two distinct stages for the lost mass of PEOZ, L-PEI, and P3HPEI. The initial decrease was caused by the loss of free and physically bound water between 30 and 150 °C; the proportion of physically bound water was approximately 2.0% for PEOZ, 4.2% for L-PEI, and 8.0% for P3HPEI. The second weight loss of these were related to thermal decomposition, and the onset of decomposition was at 390 °C for PEOZ, 380 °C for L-PEI, and 235 °C for P3HPEI, indicating that the new polymer has lower thermal stability than its parent components.

**Figure 4:**
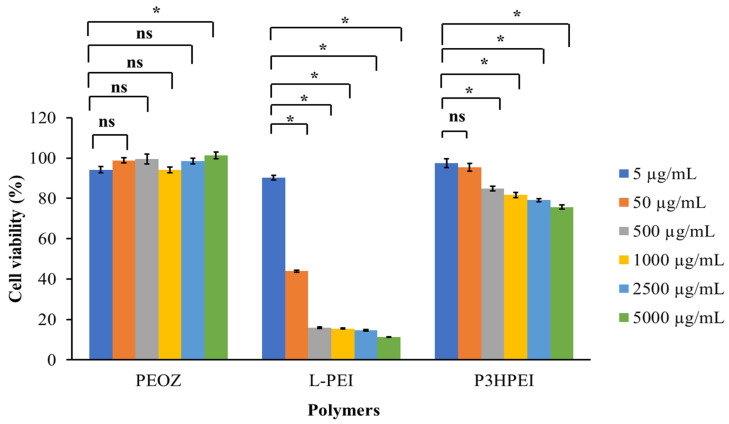
Cytotoxicity test of PEOZ, LPEI, and P3HPEI on human dermal fibroblast using an MTT assay. Statistically significant differences are given as: *—*p* < 0.05; ns—no significance.

The cytotoxicity of P3HPEI, in comparison to PEOZ and L-PEI, was assessed using an MTT assay with human dermal fibroblast cells, as shown in Figure 4. L-PEI is highly cytotoxic between 50 to 5000 µg/mL, with less than 50% cell viability when dosed at 50 µg/mL and less than 20% viability when dosed at 500 µg/mL or above. Our modification radically reduces the cytotoxicity with P3HPEI showing no significant (*p* < 0.05) effects when dosed at 5 and 50 µg/mL (viability 97.4 and 95.5%, respectively). At higher doses, a gradual decline in viability from 84.9% to 75.7% was seen as P3HPEI dosing increases from 500 to 5000 µg/mL. Consistent with the literature [36], PEOZ had no adverse effects on dermal fibroblasts as assessed by the MTT assay, with viabilities maintained above 94% for all concentrations tested. The literature contained several reports that L-PEI can cause cytotoxicity when assessed using the MTT assay [24] and that the polymer is highly cytotoxic to human dermal fibroblast cells [22]. Moghimi et al. [24] reported that L-PEI toxicity results from: (1) the disruption of cell membranes, causing necrotic cell death, and (2) the disruption of mitochondrial membranes, resulting in cell apoptosis. Furthermore, L-PEI is a cationic polymer that accumulates on the outer cell membrane, causing necrosis [37]. Fischer et al. [38] reported that the positive charge on the L-PEI surface can bind to the negative charge of cell membrane phospholipids, cell membrane proteins, and blood proteins, contributing to the interaction with the cell membrane that results in cell damage.

### 3.2. Novel Elastic Films based on Blends of Chitosan and Poly(3-hydroxypropyl ethyleneimine): Formulation, Miscibility, and Mechanical Properties

P3HPEI exhibits good solubility in water, low toxicity, and has a low glass transition temperature (−38.6 °C). Therefore, it was blended with chitosan in aqueous solutions and cast into films to improve their mechanical properties for rapid drug delivery.

**Figure 5:**
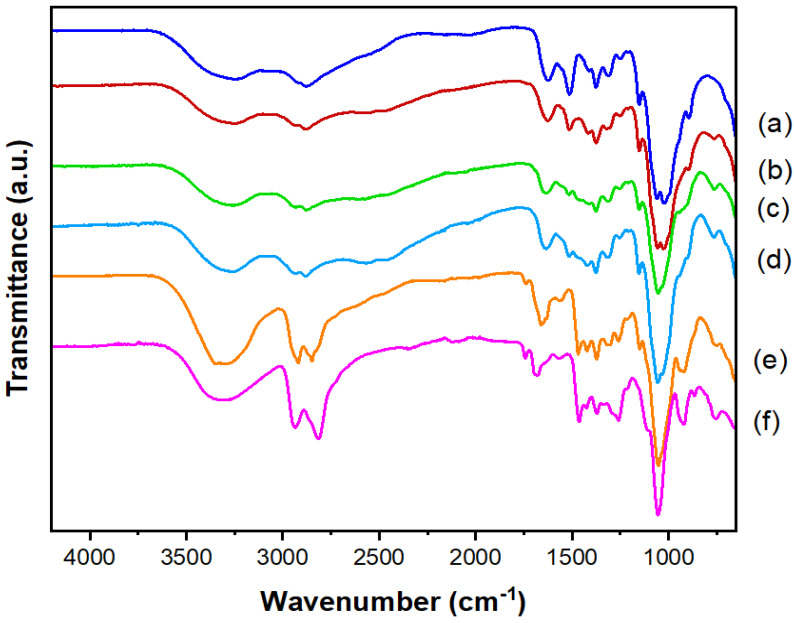
FTIR spectra of CHI (a), their blends (b–e), and P3HPEI (f). Content of P3HPEI in the blends: 20 (b), 40 (c), 60 (d), and 80% (e).

Figure 5 and Table 1 show the FTIR data from CHI films and films from blends of CHI/P3HPEI and pure P3HPEI. The FTIR spectrum of pure CHI film revealed the presence of a broad peak above 3247 cm^−1^ due to OH- stretching, which overlaps with NH- stretching in the same region. Absorption bands at 2917 and 2878 cm^−1^ correspond to -CH_2_- and -CH- stretching vibrations, respectively. The absorption bands at 1625 and 1514 cm^−1^ are consistent with C=O stretching (amide I) and NH bending (amide II). CH- and OH- vibrations give the absorption band at 1412 cm^−1^. The band at 1376 cm^−1^ represents acetamide groups, indicating that chitosan was not completely deacetylated [9], and the band at 1311 cm^−1^ was caused by C-N stretching (amide III) [39]. The band at 1250 cm^−1^ is attributable to amino groups, as reported previously [12]. The absorption bands at 1152 and 1062 cm^−1^ correspond to the anti-symmetric stretching of the C-O-C bridge and the skeletal vibrations involving the C-O stretching, which are characteristic of the chitosan polysaccharide structure [40]. The broad absorption band at 3291 cm^−1^ in the FTIR spectrum of P3HPEI likewise indicates the presence of -OH stretching, as well as bound water. Absorption bands at 2940 and 2827 cm^−1^ are attributed to -CH_2_- stretching vibrations. The absorption bands at 1464, 1371, and 1340 cm^−1^ are -CH- bending modes, whereas the absorption bands at 1297, 1260, and 1054 cm^−1^ are assigned to C-C stretching. Furthermore, P3HPEI provided an absorption band at around 1675 cm^−1^ corresponding to water, since P3HPEI is a highly viscous liquid at room temperature, supported by the -OH stretching mode at 3291 cm^−1^. All characteristic bands of the component polymers were present in the spectra of their blends, and the intensities and shapes of the bands depended on the polymer ratio in the blends. The hydroxyl region of the spectra of the miscible CHI/P3HPEI blends changed gradually, indicating a redistribution of hydroxyl group associations. In short, the -OH stretching mode shifted to higher wavenumbers (3247 to 3348 cm^−1^) as the amount of P3HPEI increased from 0 to 80%, possibly due to interactions between the polymers and water or arising from interactions between the hydroxyl and amine groups of CHI and the hydroxyl groups of P3HPEI.

**Figure 6:**
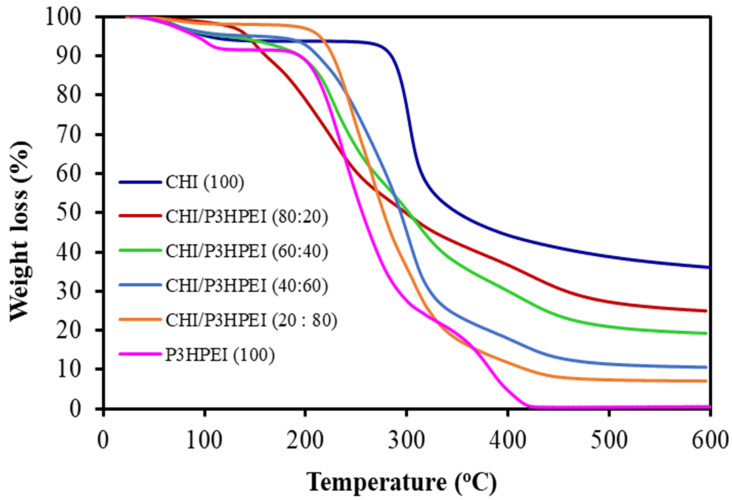
TGA thermograms of CHI film, CHI/P3HPEI blend films, and P3HPEI.

As shown in Figure 6, thermogravimetric analysis (TGA) was used to investigate the thermal stability of pure CHI film, P3HPEI, and films based on blends of CHI and P3HPEI. The results demonstrated that pure CHI film lost mass in two stages. The initial decline was due to the loss of free- and physically bound water between 30 °C and 150 °C; the proportion of physically bound water in pure CHI film was approximately 6%. The second weight loss occurred between 250 and 400 °C as the CHI film degraded by thermal decomposition; the maximum degradation rate was observed at 320 °C, resulting in a 58.3% loss in weight. Chitosan decomposes by depolymerization of its chains and pyranose rings via dehydration, deamination, and ring-opening reaction [9].

In contrast, three phases of weight loss were seen with P3HPEI. The first thermal event is evaporation of free- and bound water (approximately 8.0%) between 30 and 150 °C. Subsequently, the thermal degradation of P3HPEI is seen >235 °C (40%), followed by a further decomposition event >380 °C (89%).

The blends of CHI/P3HPEI show four weight loss events: (1) 30–150 °C, again due to loss of free and bound water, (2) 200–250 °C consistent with the early degradation of P3HPEI, (3) 275–380 °C where the later degradation of P3HPEI is seen, and (4) 350–450 °C, which correlates with the degradation of CHI. In addition, the residue of CHI/P3HPEI blends tended to decrease (34.5 to 7.1%) as the P3HPEI content in the blends increased up to 80%, with a linear correlation (R^2^ = 0.9853) confirming the miscibility of polymer blends (Figure S5).

**Figure 7:**
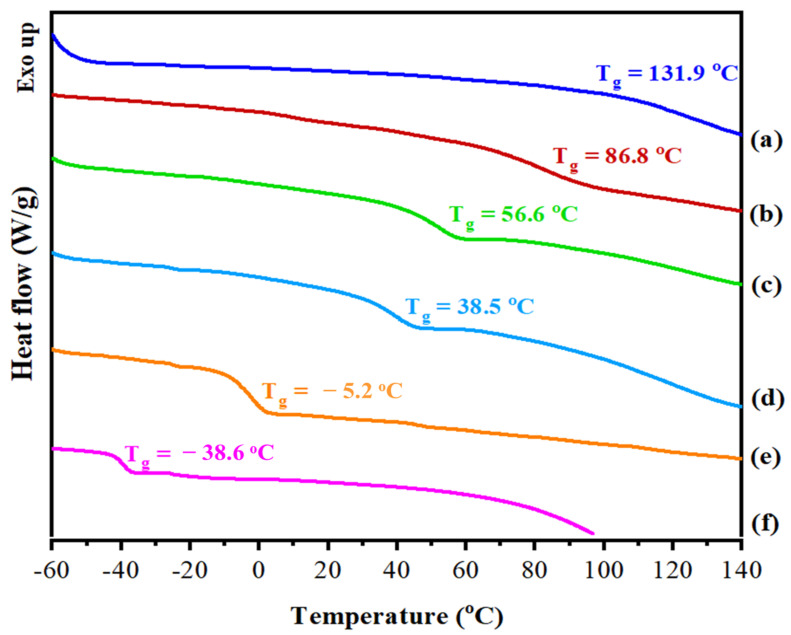
DSC thermogram of CHI (a), their blends (b–e), and P3HPEI (f). Content of P3HPEI in the blends: 20 (b), 40 (c), 60 (d), and 80% (e).

Generally, a single glass-transition temperature (T_g_) is taken as evidence for homogeneity (miscibility) in polymeric blend systems. Figure 7 shows DSC thermograms for CHI/P3HPEI blends, illustrating the presence of a single T_g_ in all compositions. As expected, the T_g_ of the CHI/P3HPEI blends all fell between the T_g_ values of the individual components (P3HPEI, −38.6 °C; chitosan, 131.9 °C) and the blends T_g_ shifted systematically to lower temperatures as the proportion of P3HPEI rose in the blends. The addition of P3HPEI, as a water soluble component, appears to have acted as a plasticizer [41,42].

**Figure 8:**
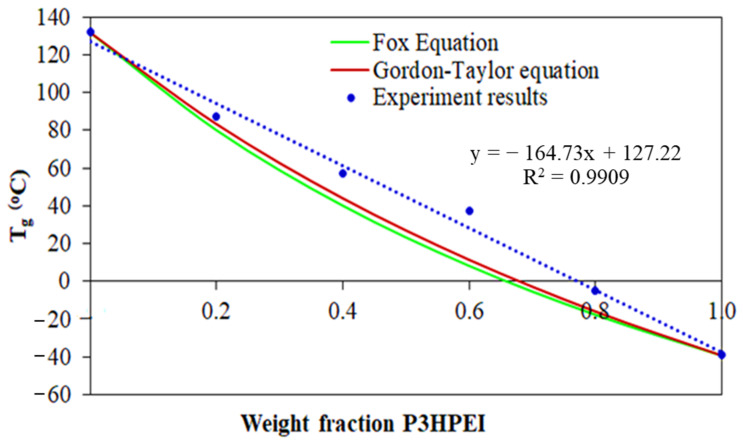
Correlation between weight fraction of P3HPEI and T_g_ of experimental results, compared with theoretical results.

Figure 8 shows the correlation between the weight fraction of P3HPEI in the blends and T_g_ using the above experimental result and those calculated theoretically; the T_g_ of miscible blends can be predicted using Fox [43] and Gordon–Taylor [44] equations, as shown in Equations (5) and (6):

(5) $$\frac{1}{\;T_{g}}=\frac{W_{\mathrm{CHI}}}{T_{g,\;\mathrm{CHI}}}+\frac{W_{P3\mathrm{HPEI}}}{T_{g,\;P3\mathrm{HPEI}}}\;\;\;\;\;\;\;\;(\mathrm{Fox}\;\mathrm{equation})$$

(6) $$T_{g}=\frac{W_{\mathrm{CHI}}T_{g,\;\mathrm{CHI}}{+\mathrm{kW}}_{P3\mathrm{HPEI}}T_{g,\;P3\mathrm{HPEI}}}{W_{\mathrm{CHI}}{+\mathrm{kW}}_{P3\mathrm{HPEI}}}\;\;\;\;(\mathrm{Gordon}–\mathrm{Taylor}\;\mathrm{equation})$$

where W_CHI_ and W_P3HPEI_ were the weight fractions of chitosan and P3HPEI, respectively; and T_g,CHI_ and T_g,P3HPEI_ are the glass transition temperatures of chitosan and P3HPEI, respectively; k is the ratio of heat capacity change in P3HPEI over chitosan [k = ΔC_p2_/ C_p1_)] [45].

The predicted glass transition temperatures of the blends were in close agreement, as shown in Figure 7, with the T_g_ decreasing as P3HPEI content increased. The experimental data sit above the theoretical curves and are commonly seen when there are interactions between the blend components [46,47,48]. The results suggest that chitosan and P3HPEI are miscible and that the chitosan and P3HPEI molecules interact, probably through intermolecular hydrogen bonds between the hydroxyl and amine groups of chitosan molecules and the hydroxyl groups of P3HPEI molecules.

Further, the interaction and miscibility of CHI and P3HPEI blends correlates with X-ray diffraction patterns from the CHI/P3HPEI films (Figure S6). CHI films show the presence of crystalline domains in this polysaccharide, which was correlated well with previous reports [9,49]. The diffraction pattern from pure P3HPEI shows no clear crystalline features but only a broad amorphous “halo”, indicating that the polymer was essentially non-crystalline. The X-ray diffractograms of CHI/P3HPEI films also reveal a broad halo, characteristic of polymers that are predominantly amorphous though some chitosan-typical diffraction peaks, could be detected in the blend films. Further, as P3HPEI content in the films increased, the sharper chitosan diffraction peaks were lost, suggesting molecular interactions between the components and their resultant miscibility.

**Figure 9:**
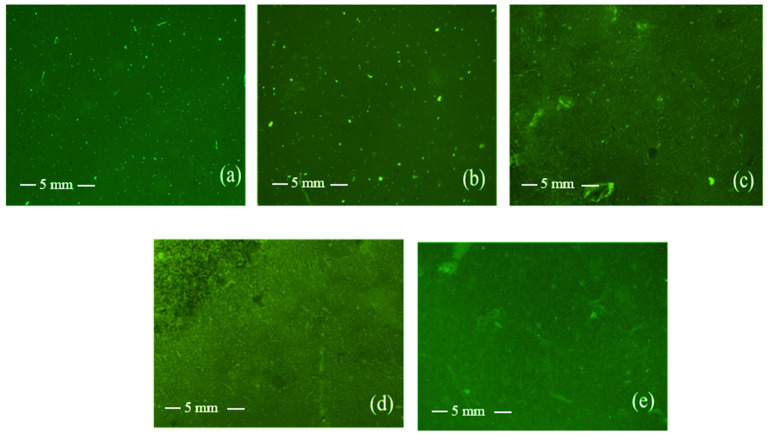
Fluorescent microscopy images of film surfaces of CHI (**a**) and their blends (**b**–**e**). Content of P3HPEI in the blends: 20 (**b**), 40 (**c**), 60 (**d**), and 80% (**e**) at 20× magnification.

Fluorescence microscopy was used to examine the morphology of the polymer film surfaces. Initially, fluorescein isothiocyanate (FITC)-labeled chitosan was synthesized and then blended with P3HPEI to form films. The images show no evidence of phase separation or interface boundaries and that all films appear homogeneous (Figure 9). Thus, the fluorescent microscope images data lend further support for miscibility of CHI and P3HPEI in the solid state at the ratios used here. The fluorescent microscope images correlated well with the scanning electron microscope (SEM) image of film surface and cross-sections (Figure S7).

**Figure 10:**
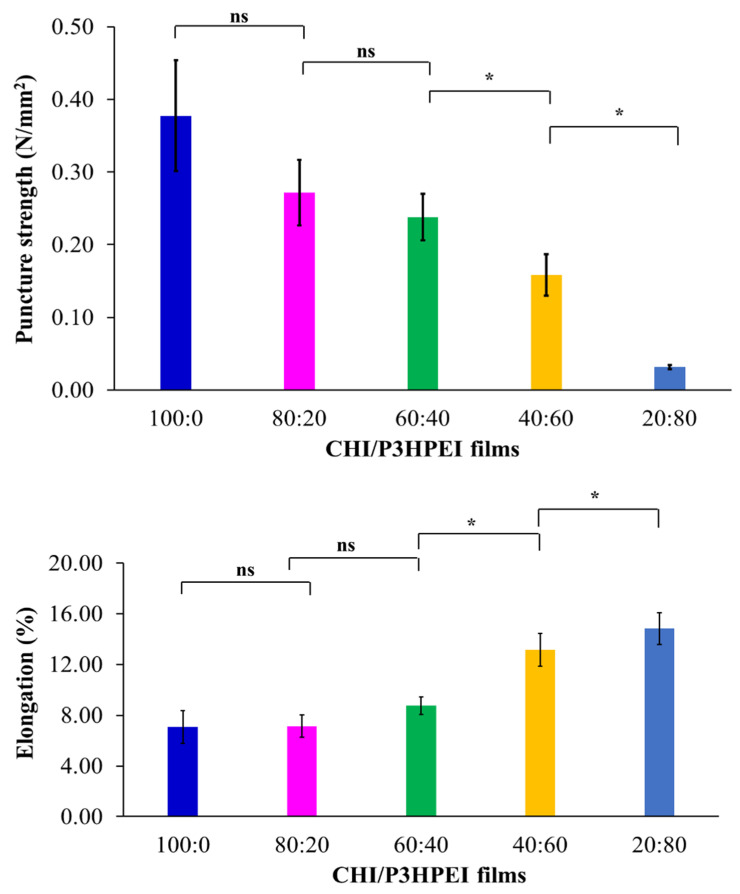

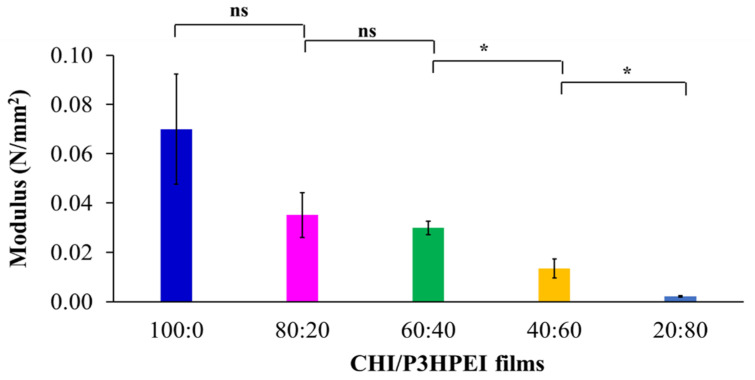
Mechanical properties of CHI and their blends with P3HPEI. Statistically significant differences are given as: *—*p* < 0.05; ns—no significance.

The mechanical properties of pure CHI film and its blends with P3HPEI are illustrated in Figure 10, including puncture strength, elongation, and modulus at puncture. Pure chitosan films (100%) had a higher puncture strength (0.38 N/mm^2^) but a lower percentage of elongation (5.62%) than the other films tested. The puncture strength of CHI/P3HPEI films decreased, compared to chitosan alone, whereas the flexibility (% elongation) increased with increasing P3HPEI content. The puncture strengths of CHI/P3HPEI films at 80:20, 60:40, 40:60, and 20:80 were 0.27, 0.24, 0.16, and 0.03 N/mm^2^, while elongation was 7.85, 7.94, 11.97, and 15.12%, respectively. The modulus at puncture was calculated from the puncture strength and elongation to predict the rigidity or stiffness of the films; the data show a similar trend to that of the puncture strength.

Clearly, increasing the P3HPEI content in the films results in more elastic materials. As shown above, P3HPEI has a low glass transition temperature (−38.6 °C) and is a water soluble polymer, so may function as a plasticizer. Plasticizers are typically relatively small molecules, such as low molecular weight polyethylene glycol (PEG), that intersperse and intercalate between polymer chains, thereby disrupting hydrogen bonding and spreading the chains apart to increase flexibility by increasing the percentage of elongation while decreasing the strength and modulus [50]. Here, increasing the P3HPEI content in CHI/P3HPEI films probably decreases intermolecular hydrogen bonding between CHI chains, resulting in enhanced chain mobility and film flexibility [51], in agreement with the thermal analysis data above.

### 3.3. Chitosan/Poly(3-hydroxypropyl ethyleneimine) Film Formulations for Loading and Delivery of Haloperidol: X-ray, Microscopic, and Drug Release Studies

Drug-loaded films have been used as drug delivery systems for administration via the transdermal, buccal, nasal, and ocular routes, which have advantages over oral and intravenous administration [52,53]. In particular, non-oral delivery methods are noninvasive or minimally invasive, painless, and simple for patients to utilize, especially those with swallowing difficulties. However, control over drug delivery is problematic with formulations, such as creams or gels [54,55]. Moreover, it was suggested that they can increase the bioavailability of various drugs [56]. Drug-loaded films have thus been developed for drug delivery systems via the topical [57], buccal [58], nasal [56], and ocular [9] routes.

Polymeric films, and, in particular, their blends, can be selected to optimize both mechanical properties and drug release [59]. The glass transition temperature (T_g_) of a polymer is an important factor when considering their use in drug delivery systems as this impacts the drug release profile; at temperatures greater than T_g_, polymer chains become flexible, and thus promote drug release [10]. Since the physiological temperature of mucosal membranes is ~35–37 °C (and skin ranges from 32 °C at the outer surface to body temperature in the inner layers) [11], a CHI/P3HPEI blend with a T_g_ ~20 °C was selected. To meet this criterion, the appropriate composition of CHI/P3HPEI was calculated from the data in Figure 7 as 35:65% (*w/w*); the T_g_ of this blend was then confirmed by DSC (Figure S8).

**Figure 11:**
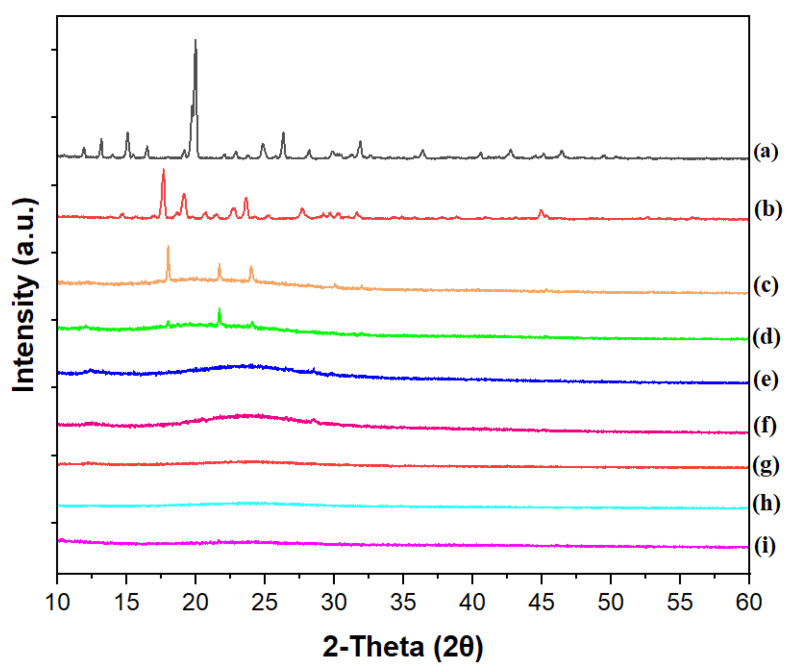
XRD diffractograms of haloperidol (a), haloperidol HCl (b), CHI/P3HPEI films loaded with haloperidol at various% drug loading, 5.0% (c), 2.5% (d), 2.0% (e), 1.75% (f), 1.5% (g), and 1.25% HP films (h) and drug-free CHI/P3HPEI film (i).

**Figure 12:**
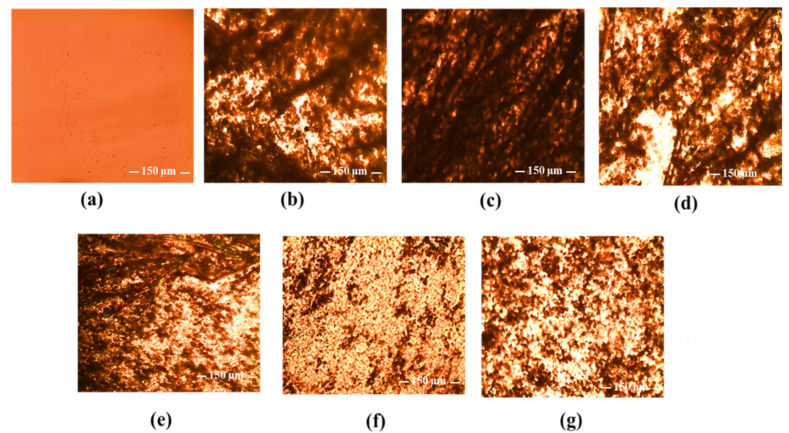
Polarized light microscope images of drug-free CHI/P3HPEI film (**a**) and CHI/P3HPEI films loaded with haloperidol (HP) at various% drug loading, 5.0% (**b**), 2.5% (**c**), 2.0% (**d**), 1.75% (**e**), 1.5% (**f**), and 1.25% (**g**) (20× magnification). Scale bars are 150 µm.

**Figure 13:**
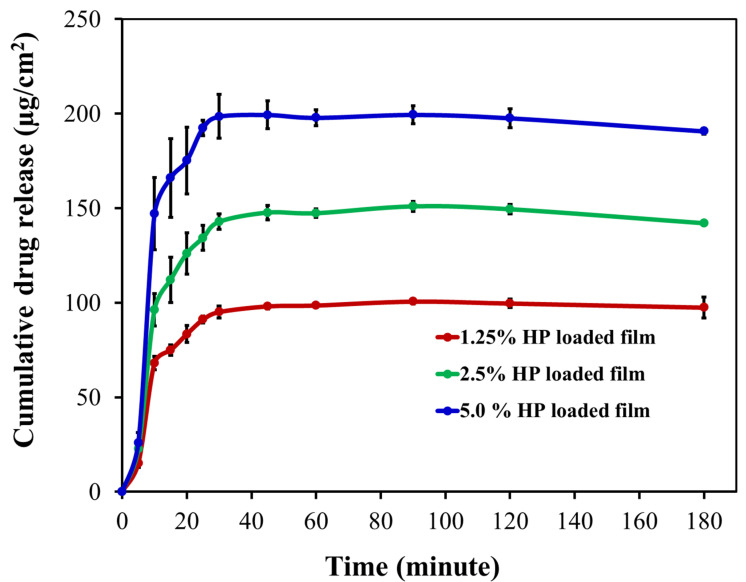
Cumulative drug release per unit area of CHI/P3HPEI films loaded with haloperidol (HP) at various % drug loading (5.0, 2.5 and 1.25%).

Haloperidol (HP) was chosen as a model poorly soluble drug for inclusion in the film patch. Generally, HP, an antipsychotic, is associated with side effects of drug-induced extrapyramidal syndrome (EPS) in conventional monotherapy [27]. HP is a BCS class 2 drug, characterized by low solubility but high permeability, and has poor oral bioavailability [29]. In this study, CHI/P3HPEI films loaded with haloperidol (HP) at various% drug loading from 1.0 to 5.0% were prepared. X-ray, microscopic, and drug-release studies of HP from CHI/P3HPEI films are shown in Figure 11, Figure 12 and Figure 13.

The X-ray diffraction (XRD) pattern of HP (Figure 11) is in agreement with literature [18]; a single crystalline polymorphic form was identified. The XRD pattern of the drug-free film showed a predominantly amorphous material with a pattern additive of those from CHI and P3HPEI. Peaks in the XRD patterns from drug-loaded films are attributable to HP crystals. However, the diffraction peaks of HP-loaded CHI/P3HPEI films exhibit a different pattern than that of HP as the free base, with peaks at 2θ = 17, 23, 25, and 31°. This is attributable to loading HP into a CHI/P3HPEI solution containing 0.1 M HCl (to solubilize chitosan), which then converts HP to its hydrochloride salt; our data is consistent with the HP-HCl reported by Al Omari et al. [60].

As expected, HP peak intensity fell with reducing drug content in the films and no crystalline materials were detected below 1.5%. This may be below the limit of detection for the instrument or, alternatively, could reflect the solubility of HP in the polymer blend.

Polarized light microscopic examination, as shown in Figure 12, provides direct visual evidence for the presence or absence of solid HP in the CHI/P3HPEI films with large crystals evident at high HP loading (2.0–5.0%), corresponding to the presence of the crystalline peaks shown by XRD. As the HP loading fell below 1.5%, the morphology of HP-loaded film altered, again reflecting the solubility of HP in the polymer blends and consistent with the XRD data. Based on these observations, HP solubility in the CHI/P3HPEI film is approximately 1.5%.

Figure 13 illustrates the cumulative release of HP (1.25, 2.5, and 5%) loaded into CHI/P3HPEI films using the Franz diffusion cell technique and a 20% PEG 400-PBS receiver solution at pH = 7.4 and 37 °C to maintain sink conditions [23]. The data show rapid release from the films, irrespective of drug loading. Drug release from films is dependent on both the physicochemical and mechanical properties of the polymers and also the nature and state of the drug, and whether encapsulated in a carrier. For the polymer matrix, the diffusion of water into the film, relaxation of the polymer chains, swelling, and erosion are important factors to consider [61,62]. Here, the hydrophilic nature of our CHI/P3HPEI films permits rapid water diffusion, swelling, and erosion whilst the low T_g_ facilitates the relaxation of the polymer chains, resulting in rapid drug release followed by disintegration of the films. In addition, release increased with drug loading, allowing dose optimization from the films. Similar loading-related release has been described by Budhian et al. [63] when HP was encapsulated in PLGA nanoparticles; here, a simple dispersion of HP in our novel polymer-blended film provides rapid release, as is desired for administration in buccal or ocular delivery.

## 4. Conclusions

Poly(3-hydroxypropyl ethyleneimine), or P3HPEI, has been successfully synthesized and its physicochemical and cytotoxic properties have been described. The polymer demonstrated good solubility in water, low toxicity, and a low glass transition temperature. P3HPEI was subsequently blended with chitosan to generate novel flexible films by casting from aqueous solutions and evaporating the solvent. The polymers in the blends were fully miscible in the solid state. Blending chitosan with P3HPEI significantly affected the elasticity and strength of films (increased elongation at the break but reduced puncture strength). A 35:65 (% *w/w*) blend of chitosan/P3HPEI provided the optimum glass transition temperature for the delivery of haloperidol through mucosal membranes. Microscopic and XRD analyses were consistent and indicated that the solubility of the drug in the films was ~1.5%. The inclusion of the hydrophilic polymer P3HPEI allowed rapid drug release followed by disintegration due to rapid water diffusion into the films, swelling, and erosion, supported by the relaxation of the polymer chains. The drug release profiles are consistent with the physicochemical properties of P3HPEI as a hydrophilic polymer with a low T_g_. Hence, blending P3HPEI with chitosan allows the selection of desirable physicochemical and mechanical properties of the films for the loading and rapid delivery of haloperidol, a model poorly water-soluble drug for transmucosal drug delivery, such as buccal or ocular administration.

## Figures and Tables

**Table 1:** FTIR absorption bands in CHI/ P3HPEI blends and their assignment.

FTIR Absorption of Blends (cm^−1^)						Assignment
100:0	80:20	60:40	40:60	20:80	0:100	
3247	3248	3258	3259	3348	3291	-OH and NH stretching
2917	2928	2929	2928	2920	2940	CH stretching
2878	2879	2880	2880	2849	2827	CH stretching
1625 ^1,2^	1624 ^1,2^	1635 ^1,2^	1631 ^1,2^	1633 ^1,2^	1675 ^2^	C=O stretching (amide I) ^1^, water region ^2^
1514 ^3,4^	1514 ^3,4^	1515 ^3,4^	1515 ^3,4^	1468 ^3,4^	1464 ^4^	NH bending (amide II) ^3^, CH_2_ vibration ^4^
1412	1416	1418	1410	1422	1424	CH and OH vibration
1376 ^5,6^	1375 ^5,6^	1376 ^5,6^	1376 ^5,6^	1372 ^5,6^	1371 ^6^	Acetamide groups ^5^, CH vibration ^6^
1311	1321	1317	1315	1302	-	CN stretching (amide III)
1152	1151	1152	1152	1150	-	Anti-symmetric strechnig of the C-O-C bridge
1060 ^7,8^	1056 ^7,8^	1055 ^7,8^	1053 ^7,8^	1053 ^7,8^	1054 ^8^	Skeletal vibration involving the C-O stretching ^7^, C-C stretching ^8^

## Data Availability

All raw data are included in Supporting Information or from the authors.

## Supplementary Materials

The following supporting information can be downloaded at: https://www.mdpi.com/article/10.3390/pharmaceutics14122671/s1, Figure S1. Synthesis scheme of P3HPEI.; Figure S2. FTIR spectra of PEOZ, LPEI and P3HPEI.; Figure S3. X-ray diffractograms of PEOZ, LPEI and P3HPEI.; Figure S4.TGA thermograms of PEOZ, LPEI and P3HPEI.; Table S1. thickness of films.; Table S2. Water loss of CHI, P3HPEI and their blends detected by TGA analysis.; Figure S5. Correlation between residue and amount of P3HPEI in CHI/P3HPEI blends.; Figure S6. X-ray diffractograms of CHI (a), their blends (b–e) and P3HPEI (f). Content of P3HPEI in the blends: 20 (b), 40 (c), 60 (d) and 80% (e).; Figure S7. SEM images of film surfaces (A) and cross section (B) of CHI (a) and their blends (b–e). Content of P3HPEI in the blends: 20 (b), 40 (c), 60 (d) and 80% (e).; Figure S8. DSC and TGA thermogram of CHI/P3HPEI (35:65).; Figure S9. FTIR of haloperidol HCl (a), haloperidol HCl with different concentrations: 5% (b), 2.5% (c), 1.5% (d) and 1.25% (e) loaded in CHI/P3HPEI films and drug free CHI/P3HPEI film (f).; Table S3. Solubility of haloperidol in various medias; Figure S10. Standard curve of haloperidol.

## References

[1] Ferreira P.G., Ferreira V.F., da Silva F.d.C., Freitas C.S., Pereira P.R., Paschoalin V.M.F. (2022). Chitosans and Nanochitosans: Recent Advances in Skin Protection, Regeneration, and Repair. Pharmaceutics.

[2] Li B., Wang J., Gui Q., Yang H. (2020). Drug-Loaded Chitosan Film Prepared via Facile Solution Casting and Air-Drying of Plain Water-Based Chitosan Solution for Ocular Drug Delivery. Bioact. Mater..

[3] Xiao C., Zhang J., Zhang Z., Zhang L. (2003). Study of Blend Films from Chitosan and Hydroxypropyl Guar Gum. J. Appl. Polym. Sci..

[4] Irikura K., Ekapakul N., Choochottiros C., Chanthaset N., Yoshida H., Ajiro H. (2021). Fabrication of Flexible Blend Films Using a Chitosan Derivative and Poly(Trimethylene Carbonate). Polym. J..

[5] Hao J.Y., Mi F.L., Shyu S.S., Wu Y.B., Schoung J.Y., Tsai Y.H., Huang Y. (2002). Bin Control of Wound Infections Using a Bilayer Chitosan Wound Dressing with Sustainable Antibiotic Delivery. J. Biomed. Mater. Res..

[6] Can A.S., Erdal M.S., Güngör S., Özsoy Y. (2013). Optimization and Characterization of Chitosan Films for Transdermal Delivery of Ondansetron. Molecules.

[7] Michailid G., OuBikiaris D.N. (2022). Novel 3D-Printed Dressings of Chitosan–Vanillin-Modified Chitosan Blends Loaded with Fluticasone Propionate for Treatment of Atopic Dermatitis. Pharmaceutics.

[8] Sogias I.A., Williams A.C., Khutoryanskiy V.V. (2008). Why Is Chitosan Mucoadhesive?. Biomacromolecules.

[9] Abilova G.K., Kaldybekov D.B., Ozhmukhametova E.K., Saimova A.Z., Kazybayeva D.S., Irmukhametova G.S., Khutoryanskiy V.V. (2019). Chitosan/Poly(2-Ethyl-2-Oxazoline) Films for Ocular Drug Delivery: Formulation, Miscibility, in Vitro and in Vivo Studies. Eur. Polym. J..

[10] Lappe S., Mulac D., Langer K. (2017). Polymeric Nanoparticles—Influence of the Glass Transition Temperature on Drug Release. Int. J. Pharm..

[11] Akash S.Z., Lucky F.Y., Hossain M., Bepari A.K., Sayedur Rahman G.M., Reza H.M., Sharker S.M. (2021). Remote Temperature-Responsive Parafilm Dermal Patch for on-Demand Topical Drug Delivery. Micromachines.

[12] Luo K., Yin J., Khutoryanskaya O.V., Khutoryanskiy V.V. (2008). Mucoadhesive and Elastic Films Based on Blends of Chitosan and Hydroxyethylcellulose. Macromol. Biosci..

[13] Yin J., Luo K., Chen X., Khutoryanskiy V.V. (2006). Miscibility Studies of the Blends of Chitosan with Some Cellulose Ethers. Carbohydr. Polym..

[14] Marsano E., Vicini S., Skopińska J., Wisniewski M., Sionkowska A. (2004). Chitosan and Poly(Vinyl Pyrrolidone): Compatibility and Miscibility of Blends. Macromol. Symp..

[15] Mohd Nasir N.F., Zain N.M., Raha M.G., Kadri N.A. (2005). Characterization of Chitosan-Poly (Ethylene Oxide) Blends as Haemodialysis Membrane. Am. J. Appl. Sci..

[16] Yeh J.T., Chen C.L., Huang K.S., Nien Y.H., Chen J.L., Huang P.Z. (2006). Synthesis, Characterization, and Application of PVP/Chitosan Blended Polymers. J. Appl. Polym. Sci..

[17] Soradech S., Williams A.C., Khutoryanskiy V.V. (2022). Physically Cross-Linked Cryogels of Linear Polyethyleneimine: Influence of Cooling Temperature and Solvent Composition. Macromolecules.

[18] Shan X., Williams A.C., Khutoryanskiy V.V. (2020). Polymer Structure and Property Effects on Solid Dispersions with Haloperidol: Poly(N-Vinyl Pyrrolidone) and Poly(2-Oxazolines) Studies. Int. J. Pharm..

[19] Mees M.A., Hoogenboom R. (2018). Full and Partial Hydrolysis of Poly(2-Oxazoline)s and the Subsequent Post-Polymerization Modification of the Resulting Polyethylenimine (Co)Polymers. Polym. Chem..

[20] Lungu C.N., Diudea M.V., Putz M.V., Grudziński I.P. (2016). Linear and Branched PEIs (Polyethylenimines) and Their Property Space. Int. J. Mol. Sci..

[21] Chatani Y., Tadokoro H., Saegusa T., Ikeda H. (1981). Structural Studies of Poly (Ethylenimine). 1. Structures of Two Hydrates of Poly (Ethylenimine): Sesquihydrate and Dihydrate. Macromolecules.

[22] Van Kuringen H.P.C., Lenoir J., Adriaens E., Bender J., De Geest B.G., Hoogenboom R. (2012). Partial Hydrolysis of Poly(2-Ethyl-2-Oxazoline) and Potential Implications for Biomedical Applications?. Macromol. Biosci..

[23] Yuan J.J., Jin R.H. (2005). Fibrous Crystalline Hydrogels Formed from Polymers Possessing a Linear Poly(Ethyleneimine) Backbone. Langmuir.

[24] Moghimi S.M., Symonds P., Murray J.C., Hunter A.C., Debska G., Szewczyk A. (2005). A Two-Stage Poly(Ethylenimine)-Mediated Cytotoxicity: Implications for Gene Transfer/Therapy. Mol. Ther..

[25] Taranejoo S., Liu J., Verma P., Hourigan K. (2015). A Review of the Developments of Characteristics of PEI Derivatives for Gene Delivery Applications. J. Appl. Polym. Sci..

[26] Patil S., Lalani R., Bhatt P., Vhora I., Patel V., Patel H., Misra A. (2018). Hydroxyethyl Substituted Linear Polyethylenimine for Safe and Efficient Delivery of SiRNA Therapeutics. RSC Adv..

[27] Samanta M.K., Dube R., Suresh B. (2003). Transdermal Drug Delivery System of Haloperidol to Overcome Self-Induced Extrapyramidal Syndrome. Drug Dev. Ind. Pharm..

[28] Abruzzo A., Cerchiara T., Luppi B., Bigucci F. (2019). Transdermal Delivery of Antipsychotics: Rationale and Current Status. CNS Drugs.

[29] Sushmita G., Maha Lakshmi J., Prathyusha K.S.S., Srinivasa Rao Y. (2018). Formulation and Evaluation of Haloperdiol-Carrier Loaded Buccal Film. Int. J. Curr. Adv. Res..

[30] Sedlacek O., Janouskova O., Verbraeken B., Richard H. (2018). Straightforward Route to Superhydrophilic Poly(2-Oxazoline)s via Acylation of Well-Defined Polyethylenimine. Biomacromolecules.

[31] Cook M.T., Tzortzis G., Charalampopoulos D., Khutoryanskiy V.V. (2011). Production and Evaluation of Dry Alginate-Chitosan Microcapsules as an Enteric Delivery Vehicle for Probiotic Bacteria. Biomacromolecules.

[32] Soradech S., Limatvapirat S., Luangtana-anan M. (2013). Stability Enhancement of Shellac by Formation of Composite Film: Effect of Gelatin and Plasticizers. J. Food Eng..

[33] Soradech S., Nunthanid J., Limmatvapirat S., Luangtana-Anan M. (2012). An Approach for the Enhancement of the Mechanical Properties and Film Coating Efficiency of Shellac by the Formation of Composite Films Based on Shellac and Gelatin. J. Food Eng..

[34] Shan X., Aspinall S., Kaldybekov D.B., Buang F., Williams A.C., Khutoryanskiy V.V. (2021). Synthesis and Evaluation of Methacrylated Poly(2-Ethyl-2-Oxazoline) as a Mucoadhesive Polymer for Nasal Drug Delivery. ACS Appl. Polym. Mater..

[35] Saegusa T., Ikeda H., Fujii H. (1972). Crystalline Polyethylenimine. Macromolecules.

[36] Lorson T., Lübtow M.M., Wegener E., Haider M.S., Borova S., Nahm D., Jordan R., Sokolski-Papkov M., Kabanov A.V., Luxenhofer R. (2018). Poly(2-Oxazoline)s Based Biomaterials: A Comprehensive and Critical Update. Biomaterials.

[37] Gholami L., Sadeghnia H.R., Darroudi M., Kazemi Oskuee R. (2014). Evaluation of Genotoxicity and Cytotoxicity Induced by Different Molecular Weights of Polyethylenimine/DNA Nanoparticles. Turkish J. Biol..

[38] Fischer D., Li Y., Ahlemeyer B., Krieglstein J., Kissel T. (2003). In Vitro Cytotoxicity Testing of Polycations: Influence of Polymer Structure on Cell Viability and Hemolysis. Biomaterials.

[39] Leceta I., Guerrero P., Ibarburu I., Dueñas M.T., De La Caba K. (2013). Characterization and Antimicrobial Analysis of Chitosan-Based Films. J. Food Eng..

[40] Silva C.L., Pereira J.C., Ramalho A., Pais A.A.C.C., Sousa J.J.S. (2008). Films Based on Chitosan Polyelectrolyte Complexes for Skin Drug Delivery: Development and Characterization. J. Membr. Sci..

[41] Matveev Y.I., Grinberg V.Y., Tolstoguzov V.B. (2000). The Plasticizing Effect of Water on Proteins, Polysaccharides and Their Mixtures. Glassy State of Biopolymers, Food and Seeds. Food Hydrocoll..

[42] Khutoryanskiy V.V., Cascone M.G., Lazzeri L., Barbani N., Nurkeeva Z.S., Mun G.A., Bitekenova A.B., Dzhusupbekova A.B. (2003). Hydrophilic Films Based on Blends of Poly(Acrylic Acid) and Poly(2-Hydroxyethyl Vinyl Ether): Thermal, Mechanical, and Morphological Characterization. Macromol. Biosci..

[43] Sakurai K., Maegawa T., Takahashi T. (2000). Glass Transition Temperature of Chitosan and Miscibility of Chitosan/Poly(N-Vinyl Pyrrolidone) Blends. Polymer.

[44] Brostow W., Chiu R., Kalogeras I.M., Vassilikou-Dova A. (2008). Prediction of Glass Transition Temperatures: Binary Blends and Copolymers. Mater. Lett..

[45] Seong D.W., Yeo J.S., Hwang S.H. (2016). Fabrication of Polycarbonate Blends with Poly(Methyl Methacrylate-Co-Phenyl Methacrylate) Copolymer: Miscibility and Scratch Resistance Properties. J. Ind. Eng. Chem..

[46] Rao V., Ashokan P.V., Shridhar M.H. (1999). Studies on the Compatibility and Specific Interaction in Cellulose Acetate Hydrogen Phthalate (CAP) and Poly Methyl Methacrylate (PMMA) Blend. Polymer.

[47] Rao V., Ashokan P.V., Shridhar M.H. (2000). Miscible Blends of Cellulose Acetate Hydrogen Phthalate and Poly(Vinyl Pyrollidone) Characterization by Viscometry, Ultrasound, and DSC. J. Appl. Polym. Sci..

[48] Mujaheddin J.R., Rai K.S., Guru G. (2012). Miscibility Studies of Agar-Agar/Starch Blends Using Various Techniques. Int. J. Res. Pharm. Chem..

[49] Shubha A., Manohara S.R., Gerward L. (2017). Influence of Polyvinylpyrrolidone on Optical, Electrical, and Dielectric Properties of Poly(2-Ethyl-2-Oxazoline)-Polyvinylpyrrolidone Blends. J. Mol. Liq..

[50] Rodríguez-Núñez J.R., Madera-Santana T.J., Sánchez-Machado D.I., López-Cervantes J., Soto Valdez H. (2014). Chitosan/Hydrophilic Plasticizer-Based Films: Preparation, Physicochemical and Antimicrobial Properties. J. Polym. Environ..

[51] Vieira M.G.A., Da Silva M.A., Dos Santos L.O., Beppu M.M. (2011). Natural-Based Plasticizers and Biopolymer Films: A Review. Eur. Polym. J..

[52] Jumelle C., Gholizadeh S., Annabi N., Dana R. (2020). Advances and Limitations of Drug Delivery Systems Formulated as Eye Drops. J. Control. Release.

[53] Jeong W.Y., Kwon M., Choi H.E., Kim K.S. (2021). Recent Advances in Transdermal Drug Delivery Systems: A Review. Biomater. Res..

[54] Latif M.S., Al-Harbi F.F., Nawaz A., Rashid S.A., Farid A., Al Mohaini M., Alsalman A.J., Al Hawaj M., Alhashem Y.N. (2022). Formulation and Evaluation of Hydrophilic Polymer Based Methotrexate Patches: In Vitro and In Vivo Characterization. Polymers.

[55] Brown M., Williams A. (2019). The Art and Science of Dermal Formulation Development.

[56] Berillo D., Zharkinbekov Z., Kim Y., Raziyeva K., Temirkhanova K., Saparov A. (2021). Stimuli-Responsive Polymers for Transdermal, Transmucosal and Ocular Drug Delivery. Pharmaceutics.

[57] Williams A., Benson H.A.E., Roberts M.S., Williams A.C., Liang X. (2021). Controlled Drug Delivery into and Through Skin. Fundamentals of Drug Delivery.

[58] Trastullo R., Abruzzo A., Saladini B., Gallucci M.C., Cerchiara T., Luppi B., Bigucci F. (2016). Design and Evaluation of Buccal Films as Paediatric Dosage Form for Transmucosal Delivery of Ondansetron. Eur. J. Pharm. Biopharm..

[59] Ahsan A., Tian W.X., Farooq M.A., Khan D.H. (2021). An Overview of Hydrogels and Their Role in Transdermal Drug Delivery. Int. J. Polym. Mater. Polym. Biomater..

[60] Al Omari M.M., Zughul M.B., Davies J.E.D., Badwan A.A. (2009). A Study of Haloperidol Inclusion Complexes with β-Cyclodextrin Using Phase Solubility, NMR Spectroscopy and Molecular Modeling Techniques. J. Solution Chem..

[61] Kraisit P., Limmatvapirat S., Luangtana-Anan M., Sriamornsak P. (2018). Buccal Administration of Mucoadhesive Blend Films Saturated with Propranolol Loaded Nanoparticles. Asian J. Pharm. Sci..

[62] Giovino C., Ayensu I., Tetteh J., Boateng J.S. (2013). An Integrated Buccal Delivery System Combining Chitosan Films Impregnated with Peptide Loaded PEG-b-PLA Nanoparticles. Colloids Surf. B Biointerfaces.

[63] Budhian A., Siegel S.J., Winey K.I. (2008). Controlling the in Vitro Release Profiles for a System of Haloperidol-Loaded PLGA Nanoparticles. Int. J. Pharm..

