# Supplementary material for: Synthesis and Evaluation of Poly(3-hydroxypropyl Ethylene-imine) and Its Blends with Chitosan Forming Novel Elastic Films for Delivery of Haloperidol
Source: Pharmaceutics. 2022 Nov 30;14(12):2671. doi: 10.3390/pharmaceutics14122671 (PMC9785711; doi:10.3390/pharmaceutics14122671)
Supplement: Supplementary file 1 [file pharmaceutics-14-02671-s001.zip › pharmaceutics-2061380-supplementary.pdf]

pharmaceutics

Supplementary information to

Synthesis and Evaluation of Poly(3-hydroxypropyl Ethylene-imine) and Its Blends with Chitosan Forming Novel Elastic Films for Delivery of Haloperidol

Sitthiphong Soradech [1,2], Pattarawadee Kengkwasingh [2], Adrian C. Williams [1] and Vitaliy V. Khutoryanskiy [1,*]

1 Reading School of Pharmacy, University of Reading, Whiteknights, Reading RG6 6DX, UK
2 Expert Centre of Innovative Herbal Products, Thailand Institute of Scientific and Technological Research, Pathum Thani 12120, Thailand
* Correspondence: v.khutoryanskiy@reading.ac.uk; Tel.: +44-(0)118-378-6119

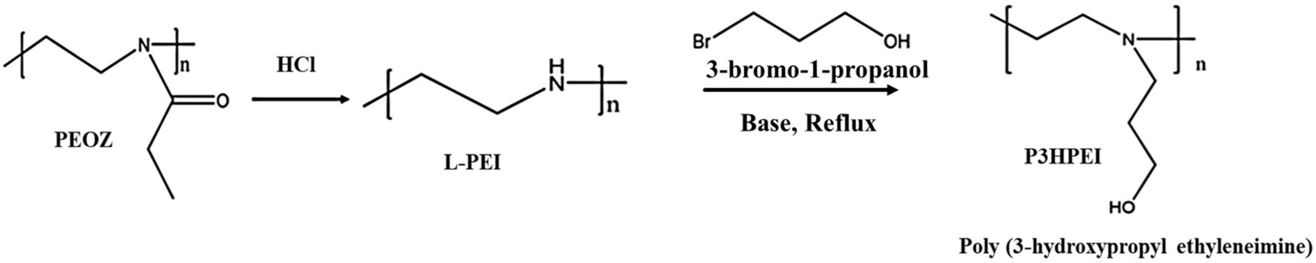

Figure S1. Synthesis scheme of P3HPEI.

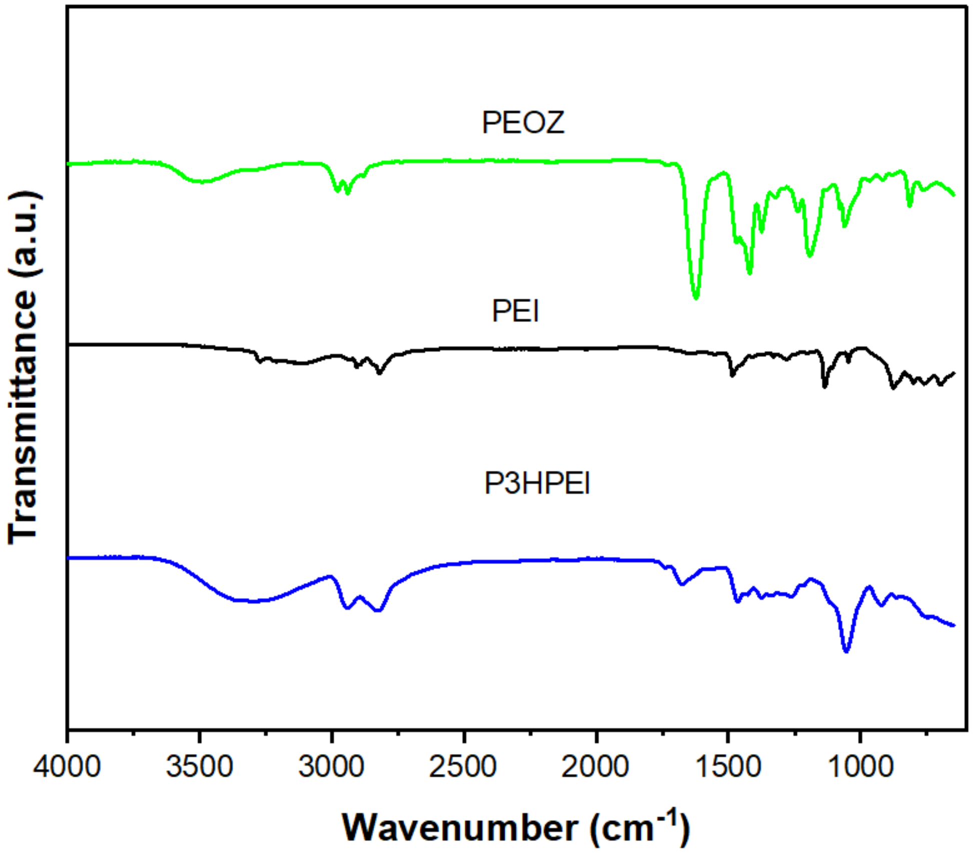

Figure S2. FTIR spectra of PEOZ, LPEI and P3HPEI.

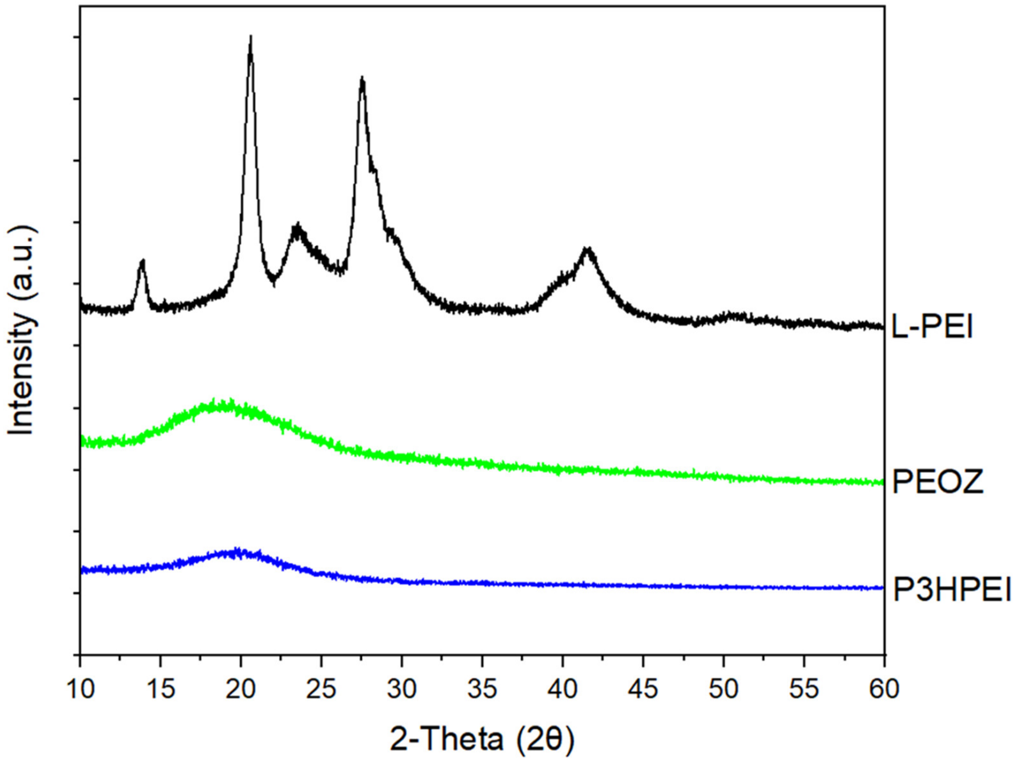

Figure S3. X-ray diffractograms of PEOZ, LPEI and P3HPEI.

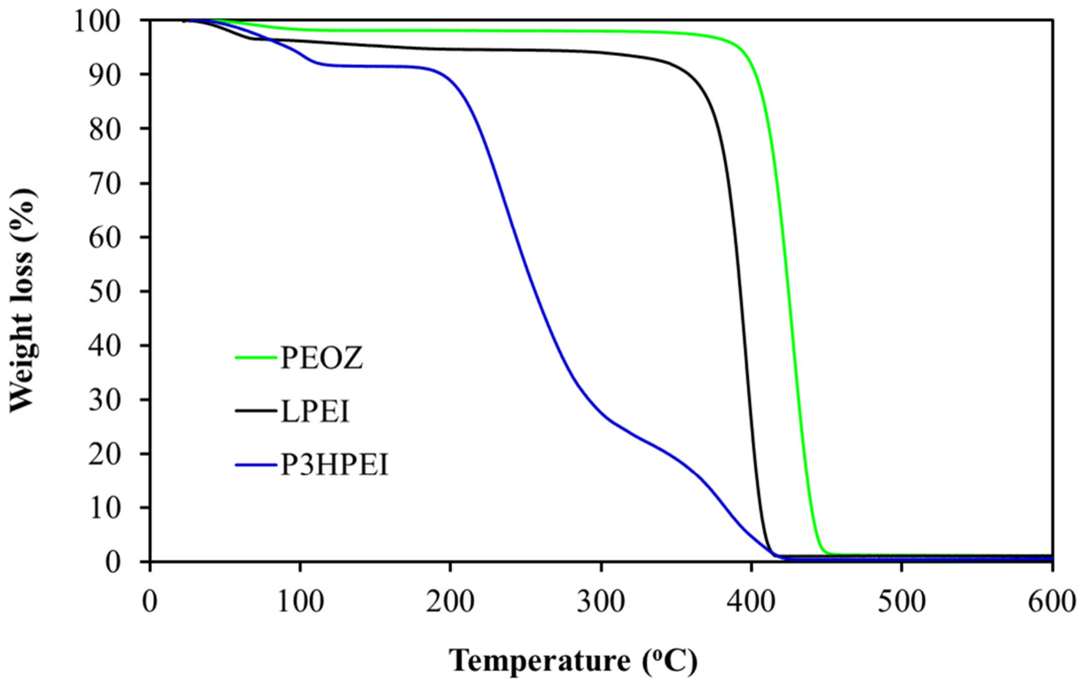

Figure S4. TGA thermograms of PEOZ, LPEI and P3HPEI.

Table S1. thickness of films.

Films	Thickness (mm)
CHI (100)	0.06 ± 0.01
CHI/P3HPEI (80:20)	0.06 ± 0.02
CHI/P3HPEI (60:40)	0.07± 0.02
CHI/P3HPEI (40:60)	0.06 ± 0.01
CHI/P3HPEI (20:80)	0.06 ± 0.01

Table S2. Water loss of CHI, P3HPEI and their blends detected by TGA analysis.

CHI/P3HPEI	Water loss (%)
CHI (100)	6.0
CHI/P3HPEI (80:20)	2.5
CHI/P3HPEI (60:40)	4.9
CHI/P3HPEI (40:60)	4.6
CHI/P3HPEI (20:80)	2.4
P3HPEI	8.0

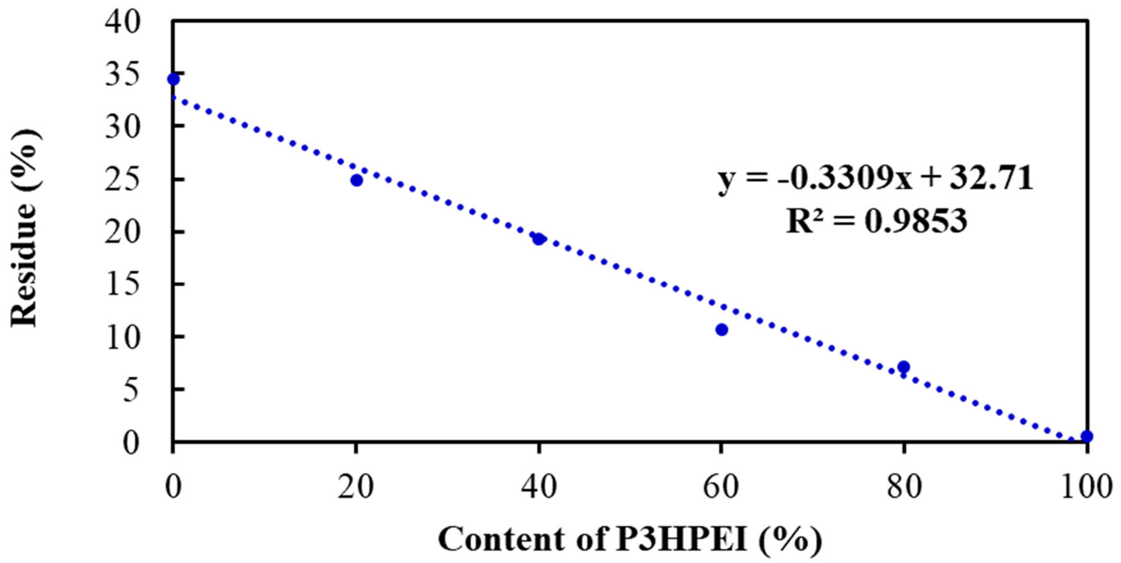

Figure S5. Correlation between residue and amount of P3HPEI in CHI/P3HPEI blends.

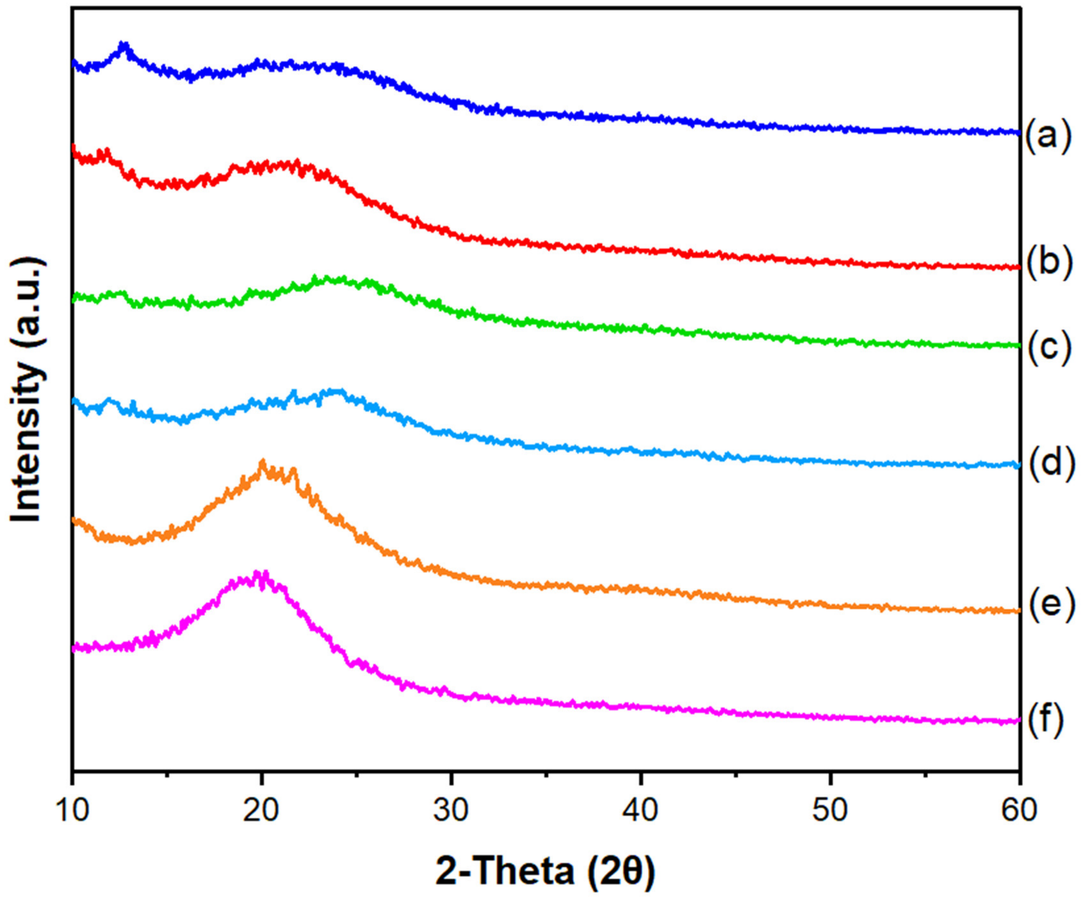

Figure S6. X-ray diffractograms of CHI (a), their blends (b, c, d and e) and P3HPEI (f). Content of P3HPEI in the blends: 20 (b), 40 (c), 60 (d) and 80 % (e).

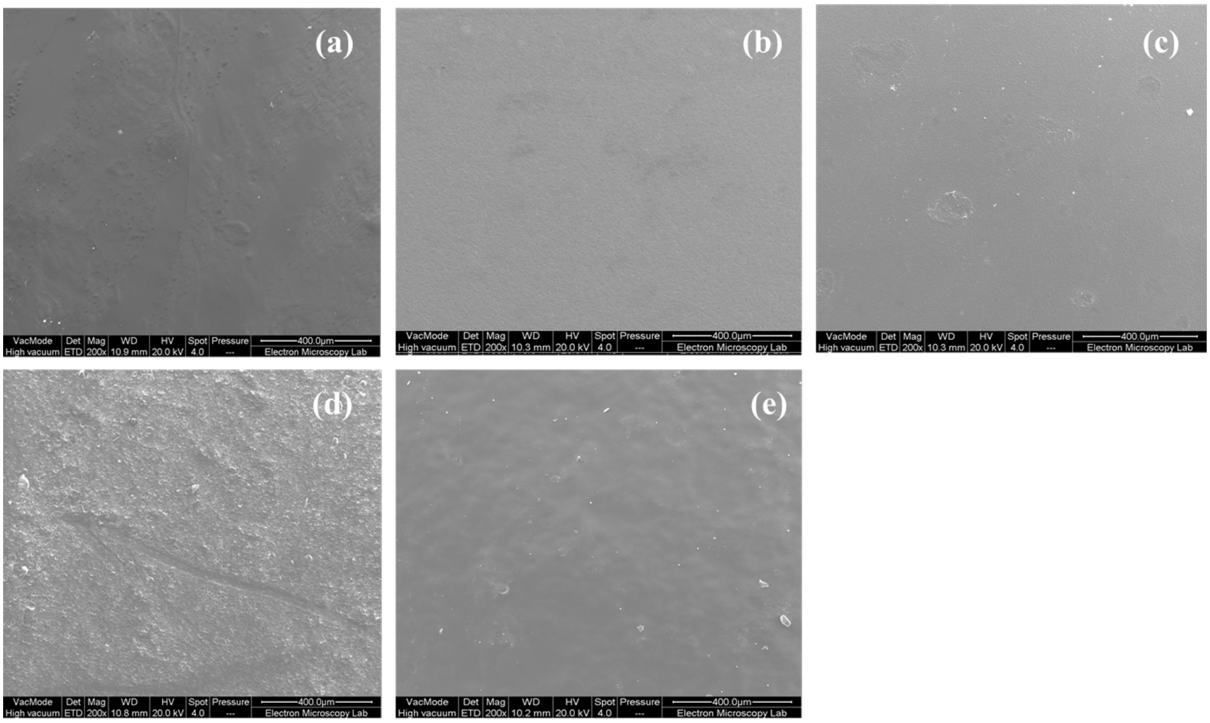

(**A**)

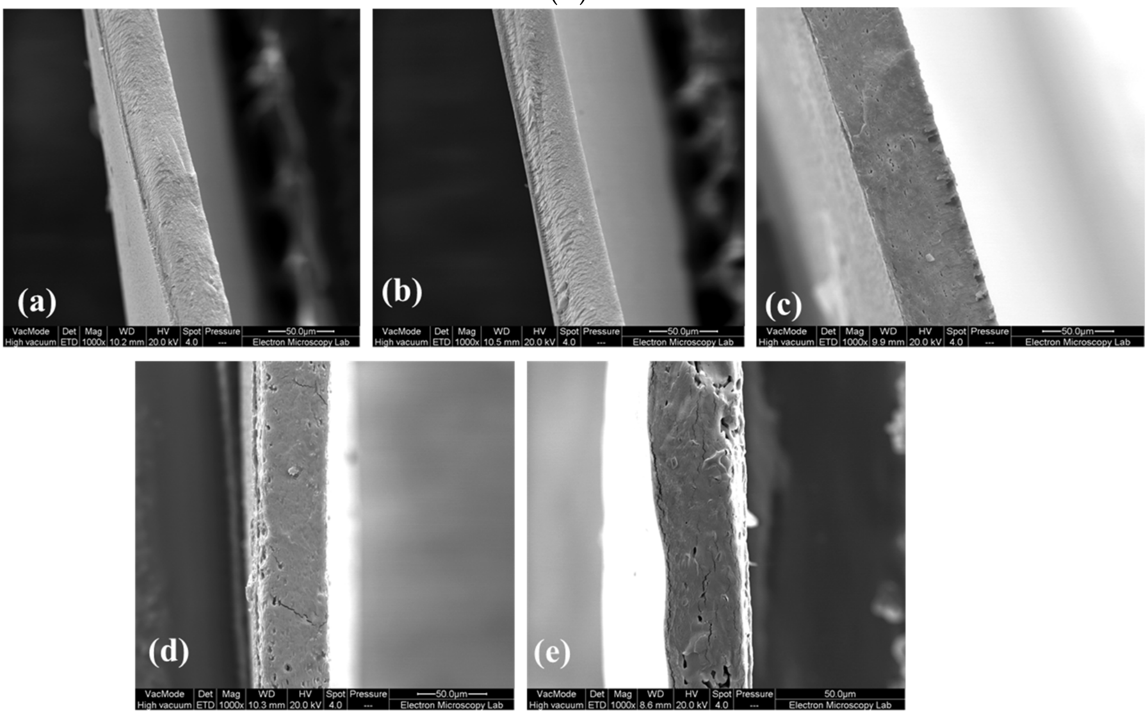

(**B**)

Figure S7. SEM images of film surfaces (**A**) and cross section (**B**) of CHI (a) and their blends (b, c, d, and e). Content of P3HPEI in the blends: 20 (b), 40 (c), 60 (d) and 80 % (e).

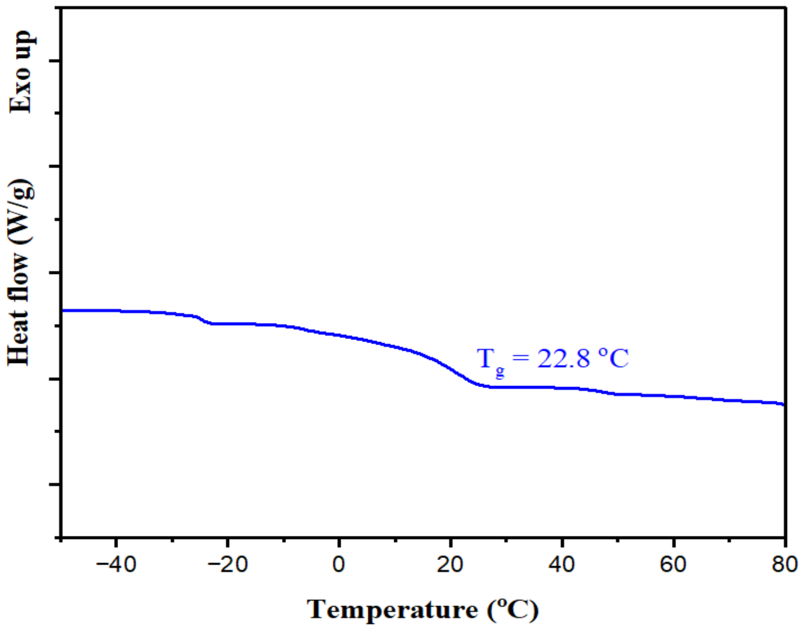

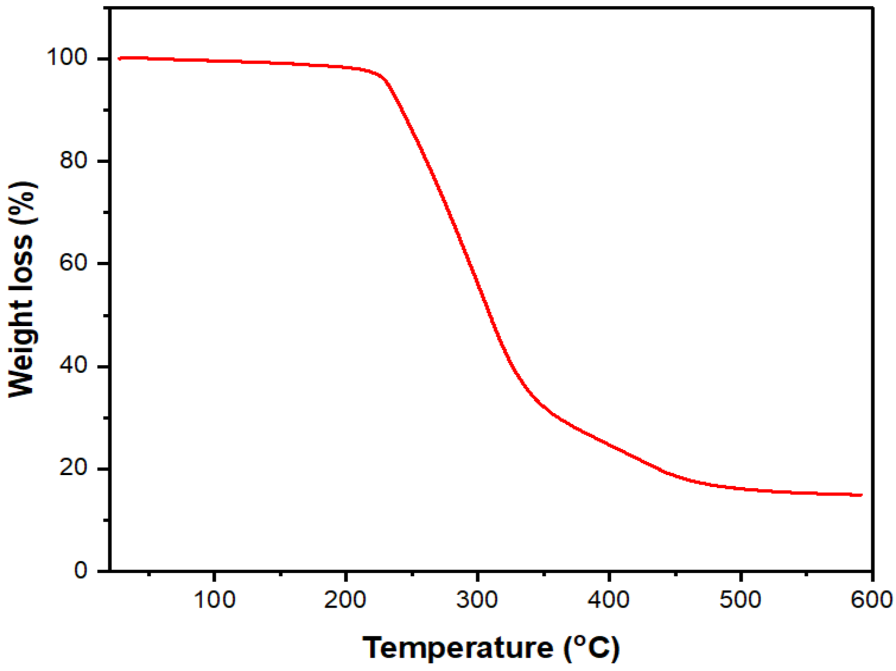

Figure S8. DSC and TGA thermogram of CHI/P3HPEI (35:65).

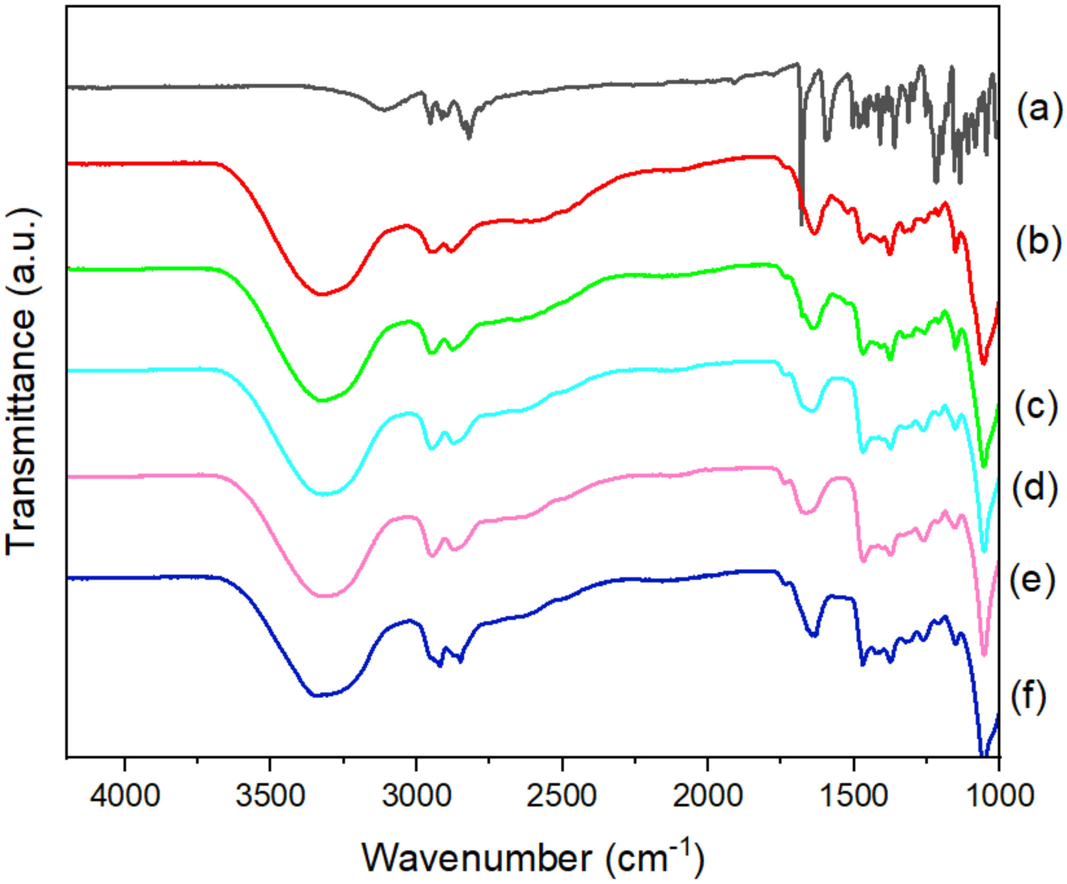

Figure S9. FTIR of haloperidol HCl (a), haloperidol HCl with different concentrations : 5% (b), 2.5% (c), 1.5% (d) and 1.25% (e) loaded in CHI/P3HPEI films and drug free CHI/P3HPEI film (f).

Table S3. Solubility of haloperidol in various media.

Medium	Solubility (mg/mL)
Distilled water	0.01
20% PEG 400-PBS pH 7.4	0.22
50% EtOH-PBS pH 7.4	0.03
95% EtOH	5.00
0.1 M HCl	3.00

Preparation of stock solution

Haloperidol (100 mg) was weighed accurately and dissolved in methanol and the volume was made up to 100 mL with the same solvent in a volumetric flask.

Preparation of Phosphate buffer solution (pH 7.4)

Phosphate buffer solution pH 7.4 was prepared by dissolving 10 tablets of Phosphate Buffered Saline in 500 mL of deionized water and then added solution into 1000 mL of volumetric flask. Subsequently, the total volume of PBS solution was adjusted to 1000 mL. The pH 7.4 of PBS was adjusted by adding 0.1 M HCl.

Preparation of working standard solutions

From the stock solution, 0.5, 1, 1.5, 2, 3, 3.5, 4.0, 4.5 and 5.0 mL were pipetted out and the volume was made up to 100 mL with 20% PEG 400 in phosphate-buffered saline (pH = 7.4) to produce concentrations of 5, 10, 15, 20, 25, 30, 35, 40, 45 and 50 µg/mL respectively. A scan was performed in order to determine the λ_{max} and the absorbance of diluted solution was measured at the λ_{max} obtained using spectrophotometer against blank buffer solution of pH 7.4 as the blank. A calibration curve (Figure S10) was constructed by plotting the absorbance against the concentration of haloperidol. A regression equation was derived from the plot, which was used for the estimation of haloperidol in 20% PEG 400 in phosphate buffer solution (pH = 7.4)

The method obeyed Beer's law in concentration range of 5 - 50 µg/mL and is suitable for the estimation of haloperidol from different sample solutions. The correlation coefficient value (r) was found to be 0.991 indicating a positive correlation between the concentration of haloperidol and the corresponding absorbance values. The regression line describes the relation between the concentration and absorbance was as follows.

$$Y = 0.0153\,X + 0.0131,$$

where, Y is the absorbance and X is the concentration of haloperidol in µg/mL

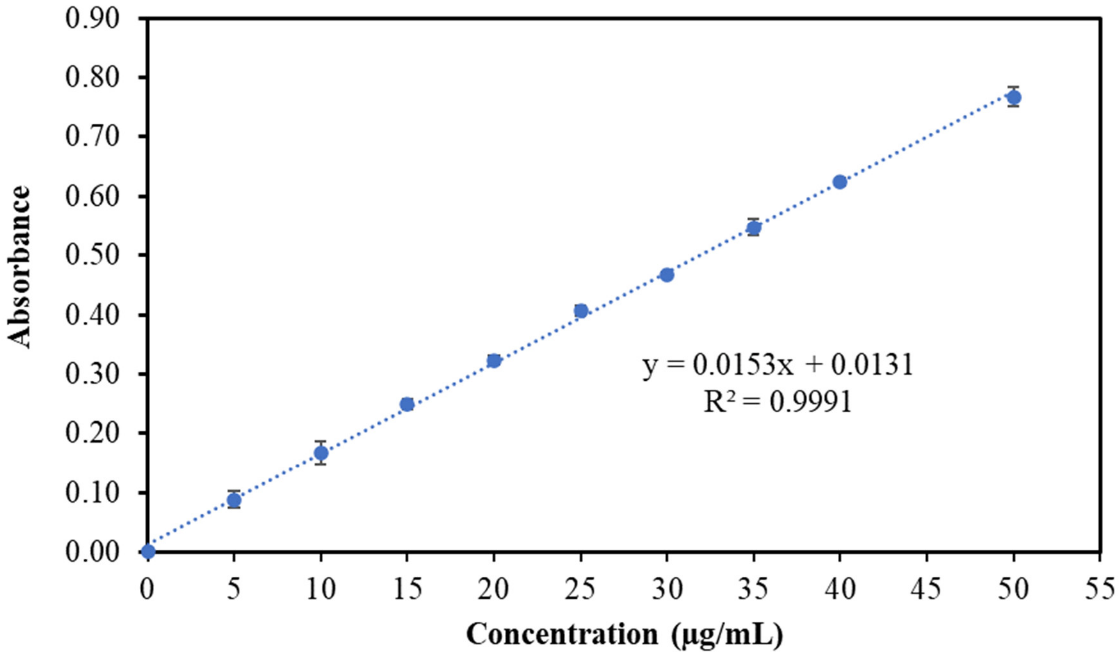

Figure S10. Calibration curve for haloperidol.
